# Morphological and Pomological Characterization of Indigenous Pear Genotypes (*Pyrus communis* L. and *P. syriaca* Boiss.) and ANN‐Based Prediction of Fruit Weight

**DOI:** 10.1002/fsn3.72401

**Published:** 2026-09-21

**Authors:** Zahra Moradi, Ali Khadivi, Yazgan Tunç

**Affiliations:** ^1^ Department of Horticultural Sciences, Faculty of Agriculture and Natural Resources Arak University Arak Iran; ^2^ Republic of Türkiye, Ministry of Agriculture and Forestry, General Directorate of Agricultural Research and Policies Hatay Olive Research Institute Directorate, Hassa Station Hassa Hatay Türkiye

**Keywords:** artificial neural network, fruit weight prediction, genetic resources, machine learning, phenotypic variation, *Pyrus* species

## Abstract

This study aimed to characterize the morphological and pomological diversity of 136 indigenous *Pyrus communis* L. and *P. syriaca* Boiss. genotypes collected from three major pear‐growing provinces of Iran and to develop an artificial neural network (ANN) model for fruit weight prediction using morpho‐pomological traits. A total of 40 quantitative and qualitative descriptors were evaluated. Phenotypic variation was analyzed using one‐way ANOVA, Pearson correlation, principal component analysis (PCA), hierarchical cluster analysis (HCA), and multiple regression analysis. A multilayer perceptron (MLP)‐based ANN model incorporating eight selected morpho‐pomological variables was developed and validated using 10‐fold cross‐validation. Significant differences were observed among genotypes for all quantitative traits (*p <* 0.05), with 87.5% of the variables exhibiting coefficients of variation above 20%, indicating substantial phenotypic diversity. Fruit weight ranged from 7.46 to 139.69 g, whereas total soluble solids varied from 10.00% to 26.00%. Fruit weight showed strong positive correlations with fruit width (*r =* 0.96), fruit length (*r =* 0.86), mesocarp diameter, and flesh thickness, while total soluble solids were negatively associated with fruit size. PCA identified three major components representing fruit size, seed morphology, and leaf serration–ripening characteristics, enabling effective discrimination among genotypes. The ANN model achieved high predictive performance for fruit weight (*R* ^2^ = 0.9739, RMSE = 5.34 g, and MAE = 4.11 g). The indigenous pear germplasm exhibited considerable morphological and pomological variation with valuable breeding potential. The integration of multivariate analyses and ANN modeling provides an effective approach for genotype evaluation and accurate fruit weight prediction, supporting the selection of superior pear genotypes for breeding and cultivar development.

## Graphical abstract

The present study demonstrated a high level of morphological and pomological variation among 136 indigenous *Pyrus communis* and *P. syriaca* genotypes. ‘Kahak‐1’ genotype had the highest fruit weight and flesh thickness, whereas ‘Razghan‐3’ genotype exhibited the highest total soluble solids content, highlighting their particular value as candidates for fresh‐market cultivars and as parents in breeding programs aiming to improve fruit size and internal quality.

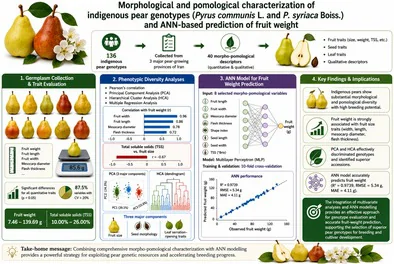

## 1 Introduction

Pear (*Pyrus* L.) is one of the most important temperate fruit crops worldwide, valued both for its economic relevance and for its nutritional contributions to the human diet, including appreciable levels of dietary fiber, sugars, organic acids, phenolic compounds, and minerals (Kolniak‐Ostek et al. 2020). West Asia, including large parts of Iran and neighboring regions, is recognized as an important centre of diversity for the genus *Pyrus*, where ancient domestication, farmer selection, and natural hybridization have generated rich assemblages of local cultivars and semi‐wild genotypes that remain only partially characterized and underutilized (Tatari et al. 2020; Parvin et al. 2025).

Morphological and pomological characterization is still the most practical and widely used tool for the initial evaluation of fruit tree genetic resources. In pear, numerous recent studies have assessed germplasm with quantitative and qualitative descriptors coupled with multivariate analyses, revealing extensive variation in key traits (fruit size, shape, skin color, soluble solids, sensory attributes) and enabling clear discrimination among local cultivars and landraces (Pereira‐Lorenzo et al. 2012; Zhang et al. 2022; Li et al. 2025; Sharma et al. 2025). In Türkiye, similar evaluations of local pear genotypes have reported broad ranges in fruit size, firmness, and biochemical composition, underlining the importance of indigenous germplasm for selecting high‐quality types adapted to regional production systems (Kan and Arpacı 2023; Kırca et al. 2023).

Understanding variation in traits that are strongly influenced by both genotypic and environmental factors and directly linked to consumer preferences—such as fruit weight, flesh texture, sweetness, grit cell content, and overall eating quality—is essential for identifying genotypes that combine superior agronomic performance with high fruit quality (Pathare and Al‐Dairi 2021; Zhang et al. 2025). Among these traits, fruit weight is particularly critical because it directly affects yield per tree, grading and packaging efficiency and market preference, and is closely associated with key morphological variables such as fruit length, fruit width, and flesh thickness, thereby contributing to overall fruit architecture (Zhang et al. 2025). Recent studies in pear and related fruit crops have shown that single‐fruit weight often exhibits among the highest coefficients of variation within pomological trait sets, highlighting its strong genetic and environmental responsiveness and considerable potential for genetic improvement (Luo et al. 2024; Waite et al. 2025). This variability provides ample scope for selecting superior genotypes and analyzing inter‐trait relationships; however, classical statistical methods based on assumptions of linearity and predictor independence (e.g., multiple linear regression) often fail to adequately capture the complex nonlinear interactions and multicollinearity that characterize morphological and quality‐related traits in long‐lived fruit trees, thereby motivating the use of more flexible machine‐learning approaches such as artificial neural networks (Sun et al. 2022).

In this context, artificial neural networks (ANNs) have emerged as a powerful tool for modeling complex relationships among horticultural traits and predicting target variables from multi‐trait datasets (Cembrowska‐Lech et al. 2023). As universal function approximators, ANNs can capture nonlinear, hierarchical relationships directly from data, making them particularly suitable for biological systems with strong interaction effects and measurement noise, as demonstrated in recent horticultural artificial intelligence (AI) research (Farooq et al. 2024).

Detailed morpho‐pomological characterization of indigenous *P. communis* L. and *P. syriaca* Boiss. genotypes combined with ANN‐based modeling for fruit weight prediction from biologically meaningful morphological and quality traits remains limited in the literature. Existing machine‐learning (ML) research in pear has focused mainly on postharvest quality monitoring and image‐based detection, leaving the application of ANNs for genotype‐level fruit weight prediction from field‐measured descriptors largely unexplored (Zhang et al. 2024; He et al. 2025). Accordingly, this study aimed (i) to comprehensively assess morpho‐pomological variation in 136 indigenous *P. communis* and *P. syriaca* genotypes from three major pear‐growing provinces of Iran (Isfahan, Kurdistan, and Markazi) using 40 quantitative and qualitative descriptors; (ii) to apply multivariate analyses to elucidate the main trait complexes underlying phenotypic variation and to identify genotypes with outstanding agronomic and fruit quality attributes; and (iii) to develop and evaluate an ANN model for predicting fruit weight from statistically and biologically relevant morpho‐pomological traits, and to compare its performance with conventional multivariate analysis.

## 2 Materials and Methods

### 2.1 Plant Material

**FIGURE 1:**
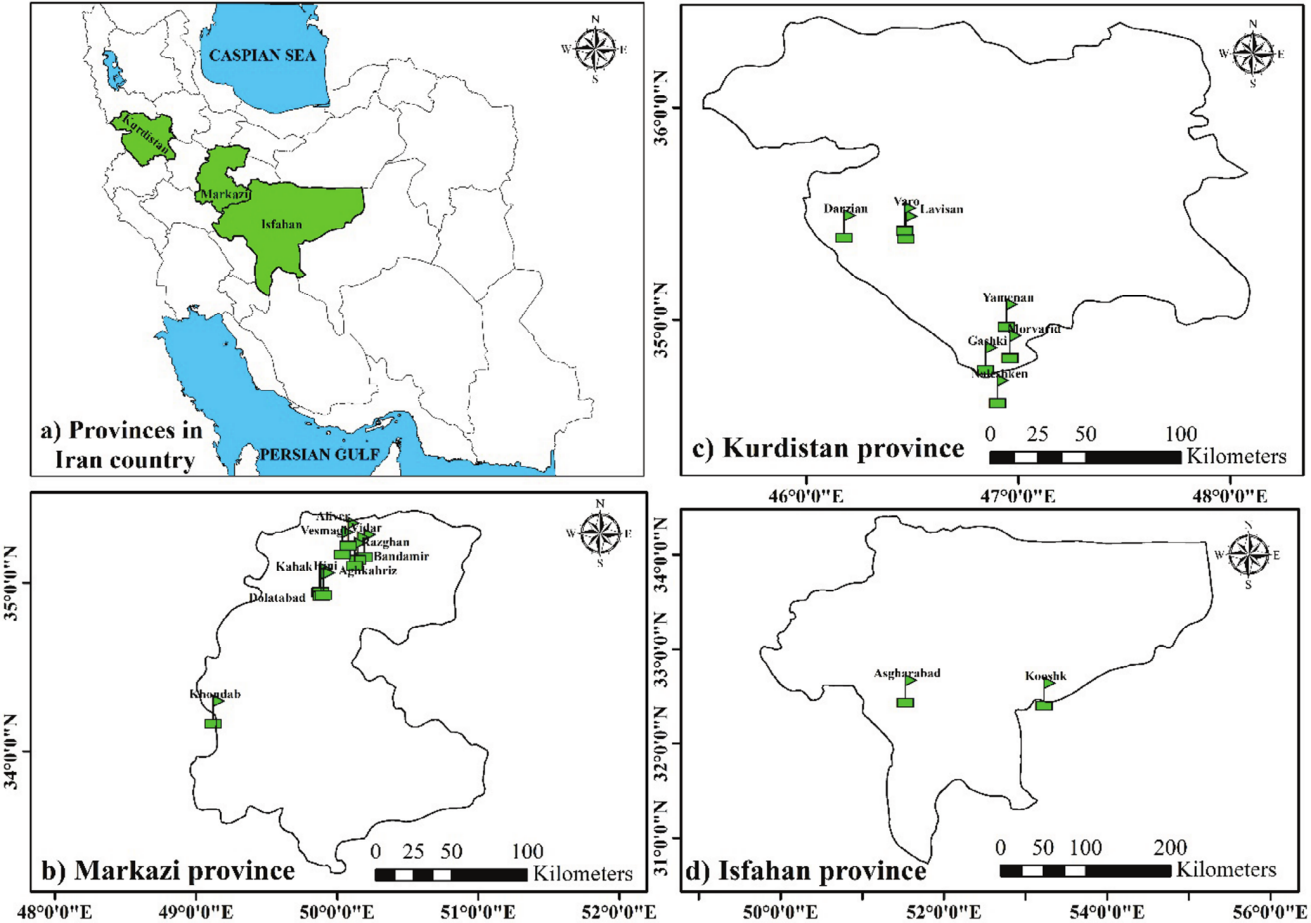
Geographic locations of collection sites of the studied pear genotypes. The above map image is original and was generated by the second author, Ali Khadivi, using ArcGIS software version 10.1 (http://www.arcgis.com/home/item.html?id=30e5fe3149c34df1ba922e6f5bbf808f).

Morphological variation among 136 indigenous genotypes belonging to two pear species (111 *P. communis* and 25 *P. syriaca* ) was evaluated in several regions of Isfahan, Kurdistan, and Markazi provinces, Iran. The genotypes collected from Tolaee, Kaftarbache, Gonjooni, Hini, Kahak, Vesmagh, Dolatabad, Aliver, Vidar, Aghkahriz, Razghan, Bandamir, and Khondab regions belonged to *P. communis* , whereas the genotypes collected from Kamyaran and Marivan regions belonged to *P. syriaca* . Geographic locations of collection sites of the studied genotypes are shown in Table S1 and Figure 1. The map in Figure 1 was generated using ArcGIS software version 10.1 (ESRI 2012).

Each genotype included in this study was represented by a single naturally occurring tree growing in the wild. Consequently, a total of 136 individual trees (111 *P. communis* and 25 *P. syriaca* ) were sampled, with each tree representing a distinct genotype. Because the study focused on naturally occurring indigenous germplasm rather than cultivated accessions, replicate trees of the same genotype were not available for sampling.

The unequal number of genotypes between *P. communis* (111 genotypes) and *P. syriaca* (25 genotypes) reflects their natural distribution and field availability in the surveyed regions rather than a predefined sampling scheme. Because the objective of this study was to comprehensively characterize the indigenous pear germplasm, all naturally occurring and eligible genotypes encountered during field exploration were included. Consequently, no attempt was made to artificially balance the number of genotypes between species, thereby preserving the natural representation of the available genetic diversity.

To reduce the likelihood of inadvertently sampling clonally propagated individuals, sampled trees within each site were spaced at least 200 m apart. To further ensure that sampled individuals represented distinct genets rather than vegetatively connected ramets, we additionally applied the following criteria: (i) sampling was restricted to naturally regenerated wild populations; (ii) individuals showing evidence of vegetative propagation (e.g., root suckering, multi‐stem clonal clumps, or apparent belowground connections) were excluded; and (iii) only well‐isolated trees with clearly independent canopy architectures and growth forms were selected.

### 2.2 Morphological Evaluations

In total, 40 morphological and pomological variables were used to assess phenotypic variability among the selected genotypes (Table 1). Morphological and pomological assessments were performed using 50 leaf and 50 fruit samples per genotype. Leaf length, leaf width, petiole length, petiole width, fruit length, fruit width, fruit stalk length, fruit stalk diameter, endocarp diameter, mesocarp diameter, fruit flesh thickness, seed length, seed width, and seed thickness were measured using a digital caliper (Mitutoyo ABSOLUTE Digimatic, CD‐15APX series, Mitutoyo Corp., Japan) with a resolution of 0.01 mm and a manufacturer‐specified accuracy of ±0.02 mm. Fruit weight was determined using an electronic balance (Radwag AS 220.R2, RADWAG, Poland) with a precision of 0.01 g. The remaining traits were recorded as qualitative descriptors using rating scales and codes (Table 2), following the standardized pear descriptors published by IPGRI/CEC (International Plant Genetic Resources Institute; CEC and IPGRI 1983). To minimize observer bias, all qualitative assessments were performed jointly by the same two trained evaluators.

**TABLE 1:** Descriptive statistics of the morphological and pomological traits evaluated in the studied pear genotypes.

Trait	Unit	Abbr.	Min	Max	Mean	± SD	CV (%)	*p*	Significance
Leaf shape	Code	V1	1	3	2.79	0.61	21.86	—	—
Leaf shape base	Code	V2	1	3	1.24	0.65	52.18	—	—
Leaf color	Code	V3	1	5	3.13	1.15	36.65	—	—
Leaf margin dentation (Presence of teeth)	Code	V4	0	1	0.91	0.29	31.32	—	—
Leaf serration depth	Code	V5	0	5	1.49	1.03	69.33	—	—
Leaf serration shape	Code	V6	0	3	2.71	0.88	32.44	—	—
Petiole color	Code	V7	1	15	4.75	3.08	64.91	—	—
Leaf length	mm	V8	40.38	74.78	56.34	6.75	11.98	0.003	**
Leaf width	mm	V9	14.57	48.74	30.74	6.73	21.91	0.018	*
Petiole length	mm	V10	18.61	56.40	37.75	7.19	19.05	0.001	**
Petiole diameter	mm	V11	0.39	1.41	0.92	0.18	19.98	0.032	*
Ripening date	Code	V12	1	13	10.29	2.12	20.56	—	—
Fruit shape	Code	V13	1	5	2.81	0.68	24.31	—	—
Fruit symmetry	Code	V14	0	1	0.19	0.40	207.89	—	—
Fruit tip shape	Code	V15	1	3	1.88	1.00	53.03	—	—
Fruit skin ground color	Code	V16	1	9	4.60	2.96	64.35	—	—
Fruit skin over color	Code	V17	1	7	2.34	1.77	75.77	—	—
Fruit skin russet	Code	V18	0	5	0.86	1.16	134.42	—	—
Color of spots on fruit skin	Code	V19	1	7	3.99	1.09	27.29	—	—
Fruit length	mm	V20	18.80	61.10	43.01	9.40	21.85	0.001	**
Fruit width	mm	V21	23.80	68.73	43.81	9.94	22.68	0.001	**
Fruit weight	g	V22	7.46	139.69	51.87	29.85	57.55	0.001	**
Fruit stalk length	mm	V23	14.48	73.31	39.60	9.38	23.69	0.009	**
Fruit stalk diameter	mm	V24	1.52	5.03	2.73	0.50	18.32	0.021	*
Endocarp diameter	mm	V25	6.70	33.83	16.73	4.04	24.17	0.004	**
Mesocarp diameter	mm	V26	17.81	62.52	41.64	10.06	24.17	0.002	**
Fruit flesh thickness	mm	V27	5.33	24.92	13.66	4.51	33.02	0.001	**
Total soluble solids	%	V28	10.00	26.00	17.21	3.17	18.44	0.007	**
Fruit flesh color	Code	V29	1	9	3.68	2.41	65.57	—	—
Fruit flesh firmness	Code	V30	1	5	3.24	1.48	45.74	—	—
Grit cell amount	Code	V31	1	5	2.26	1.33	58.81	—	—
Grit cell size	Code	V32	1	5	3.94	1.42	35.99	—	—
Fruit taste	Code	V33	1	11	6.38	2.86	44.75	—	—
Fruit quality	Code	V34	1	5	2.62	1.62	61.98	—	—
Fruit breakdown	Code	V35	1	5	2.63	1.81	68.78	—	—
Seed number per fruit	Number	V36	3	15	8.16	2.12	25.99	0.041	*
Seed length	mm	V37	3.5	11.53	8.52	1.73	20.29	0.001	**
Seed width	mm	V38	1.12	13.26	4.80	1.61	33.49	0.015	*
Seed thickness	mm	V39	0.12	14.18	3.02	1.50	49.67	0.006	**
Seed color	Code	V40	1	5	3.10	1.64	52.87	—	—

Abbreviations: ± SD, standard deviation; CV, coefficient of variation; Max, maximum; Min, minimum.

*p < 0.05, **p < 0.01.

**TABLE 2:** Frequency distribution of the qualitative morphological traits evaluated in the studied pear genotypes.

Trait	Frequency (no. of genotypes)								
	0	1	3	5	7	9	11	13	15
Leaf shape	—	Lanceolate (14)	Oval (122)	—	—	—	—	—	—
Leaf shape base	—	Acute (120)	Round (16)	—	—	—	—	—	—
Leaf color	—	Light green (18)	Green (91)	Dark green (27)	—	—	—	—	—
Leaf margin dentation (Presence of teeth)	Absent (12)	Present (124)	—	—	—	—	—	—	—
Leaf serration depth	Absent (12)	Low (86)	Intermediate (37)	High (1)	—	—	—	—	—
Leaf serration shape	Absent (12)	Smooth (2)	Serrate (22)	—	—	—	—	—	—
Petiole color	—	White (12)	White‐green (68)	Light white (20)	Yellow‐green (16)	Yellow‐white (8)	Yellow (6)	Yellow‐red (5)	White‐red (1)
Ripening date	—	Mid‐August (2)	Late August (1)	Early September (2)	Mid‐September (1)	Late September (53)	Early October (50)	Mid‐October (27)	—
Fruit shape	—	Pear‐shaped (15)	Quince‐shaped (119)	Apple‐shaped (2)	—	—	—	—	—
Fruit symmetry	Absent (110)	Present (26)	—	—	—	—	—	—	—
Fruit tip shape	—	Flat (76)	Round (60)	—	—	—	—	—	—
Fruit skin ground color	—	Light green (39)	Green (21)	Yellow‐green (30)	Yellow (20)	Lemon‐yellow (26)	—	—	—
Fruit skin over color	—	Light green (81)	Yellow (21)	Lemon‐yellow (32)	Orange‐red (2)	—	—	—	—
Fruit skin russet	Absent (65)	Low (55)	Intermediate (14)	High (4)	—	—	—	—	—
Color of spots on fruit skin	—	White (1)	Yellow (69)	Light green (64)	Orange‐red (2)	—	—	—	—
Fruit flesh color	—	White (35)	Cream (60)	Yellow (9)	White‐green (24)	White‐yellow (8)	—	—	—
Fruit flesh firmness	—	Low (30)	Intermediate (60)	High (46)	—	—	—	—	—
Grit cell amount	—	Low (64)	Intermediate (58)	High (14)	—	—	—	—	—
Grit cell size	—	Small (17)	Intermediate (38)	Large (81)	—	—	—	—	—
Fruit taste	—	Tasteless (17)	Sour (4)	Sour–sweet (39)	Slightly sweet (32)	Sweet (32)	Very sweet (12)	—	—
Fruit quality	—	Low (60)	Intermediate (42)	High (34)	—	—	—	—	—
Fruit breakdown	—	Low (70)	Intermediate (21)	High (45)	—	—	—	—	—
Seed color	—	Brown (42)	Dark brown (45)	Black (49)	—	—	—	—	—

Total soluble solids content of the fruits was determined using a digital refractometer (Atago PAL‐1, Atago Co. Ltd., Tokyo, Japan). For each genotype, juice was extracted from the equatorial region of freshly harvested fruits after removing the peel and seeds. Before measurement, the refractometer was calibrated with distilled water at 20°C. A few drops of clear fruit juice were placed on the prism surface, and total soluble solids values were recorded as °Brix. Measurements were performed on 20 fruits per genotype, and the mean value was used for statistical analyses.

Although 50 fruits per genotype were used for morphological evaluations, TSS was determined using juice extracted from 20 representative fruits. This sample size is widely considered sufficient to estimate genotype‐level soluble solids content because TSS generally exhibits lower within‐tree variability than morphological fruit traits while requiring destructive laboratory analysis. Therefore, 20 representative fruits provided a reliable estimate of the mean TSS for each genotype.

### 2.3 Statistical Analysis

This study was conducted over two consecutive growing seasons (2023 and 2024) using the same permanently identified individual genotypes. As each genotype was represented by a single naturally occurring individual in the field, all measurements were performed on that individual. For each season, 50 biological samples of leaves, fruits, and seeds were randomly collected from each genotype and measured separately. Initially, organ‐level measurements were averaged to obtain mean values for each genotype. These means were then averaged within each year to calculate annual means. Finally, the annual means from 2023 and 2024 were combined to derive the final two‐year means used in all subsequent statistical analyses.

A one‐way analysis of variance (ANOVA) was performed at the 5% significance level (*p <* 0.05) using the JMP Pro 17 statistical package (JMP 2024). Relationships among the measured traits were evaluated using Pearson correlation coefficients (*r*) (Pearson 1900), calculated with Origin Pro 2025b software (OriginLab 2025). Principal component analysis was applied in the same software environment to identify the most influential variables contributing to genotypic differentiation. To enhance the interpretability of factor loadings, Varimax rotation with Kaiser normalization was employed (Kaiser 1958, 1960), and only components with eigenvalues ≥ 1, in accordance with the Kaiser criterion, were retained.

To classify genotypes into distinct groups, hierarchical cluster analysis was conducted using Ward's linkage method (Ward Jr. 1963) in combination with Euclidean distance measures in Origin Pro 2025b. Before clustering, the dataset was preprocessed through min–max scaling (rescaling to a 0–1 range) followed by *Z*‐score normalization to minimize bias arising from differences in measurement scales and to ensure comparability among variables (Hammer et al. 2001). The optimal number of clusters was determined by examining the dendrogram and selecting the cut‐off point corresponding to the largest increase in linkage distance (fusion distance), consistent with Ward's minimum variance criterion. The resulting clustering pattern was further evaluated by comparison with the distribution of genotypes in the PCA biplot. A biplot of the first two principal components (PC1 and PC2) was subsequently generated to visualize the distribution of genotypes and their associations with traits in a reduced‐dimensional space. In addition, principal component score analysis (PCSA) were calculated for each genotype based on the principal component analysis model of pear genotypes, providing a concise representation of multidimensional variation and highlighting structural differences among samples.

Composite PCSA were calculated to provide an overall ranking of the genotypes based on the principal components retained after PCA. Only principal components with eigenvalues ≥ 1.0 (Kaiser criterion) were included in the calculation. The composite score for each genotype was obtained as the weighted sum of the retained principal component scores, where the weight assigned to each component corresponded to its proportion of explained variance. The composite PCSA was calculated as (1):

(1) $$\text{PCSA}=\sum_{i=1}^{k}\left({\mathrm{PC}}_{i}\times {\mathrm{EV}}_{i}\right)$$

where PC_*i*_ is the standardized score of the *i*th principal component, EV_*i*_ is the proportion of variance explained by that component (expressed as a decimal), and k is the number of retained principal components. Genotypes with higher composite PCA scores were considered to exhibit more extreme or favorable multivariate trait profiles. Furthermore, to investigate the effects of other variables on selected fruit traits, which were considered as dependent variables, stepwise multiple linear regression analysis was performed using SPSS software (SPSS Inc., Chicago, IL, USA), following the statistical procedures described by Norusis (1998), Efe et al. (2000), and SPSS (2012).

### 2.4 Artificial Neural Network (ANN) Modeling

An artificial neural network (ANN) model was developed to predict fruit weight using morpho‐pomological traits of pear genotypes. A feed‐forward multilayer perceptron (MLP) architecture was employed to capture potential nonlinear relationships among traits (Basheer and Hajmeer 2000; Khaki and Wang 2019; Kuan 2018). Selection of the ANN input variables followed a three‐stage procedure combining statistical screening and biological interpretation. First, Pearson's correlation analysis and stepwise multiple linear regression were used to identify traits showing significant associations with fruit weight (*p <* 0.05) and meaningful predictive contributions. Principal component analysis (PCA) was then examined to identify variables with high loadings on the major components explaining phenotypic variation. Finally, among the statistically supported variables, biologically meaningful traits directly related to fruit development and fruit size were retained while avoiding redundant predictors with overlapping biological information. Based on this procedure, eight variables (fruit width, fruit length, seed width, fruit tip shape, leaf width, fruit flesh thickness, grit cell amount, and fruit stalk diameter) were selected as the ANN input variables.

The optimal network architecture was determined empirically using an iterative trial‐and‐error procedure. Several candidate MLP models with different numbers of hidden neurons were trained and evaluated using 10‐fold cross‐validation. Model performance was compared according to the coefficient of determination (*R* ^2^), root mean squared error (RMSE), and mean absolute error (MAE). The architecture providing the highest predictive accuracy together with the lowest prediction errors, while maintaining good generalization ability and minimizing the risk of overfitting, was selected as the final model.

The ANN employed in this study followed a conventional feed‐forward MLP architecture consisting of an input layer (8 neurons), a single hidden (pattern) layer (5 neurons), and an output layer (1 neuron). During forward propagation, the weighted outputs of the hidden neurons were combined through a summation operation before being passed to the output neuron. Thus, the term “summation layer” refers to this computational aggregation step rather than an additional trainable layer. All neurons were fully connected through weighted synaptic connections.

Before network training, all predictor variables were standardized using *Z*‐score normalization (mean = 0, standard deviation = 1) to eliminate scale effects and improve numerical stability. Fruit weight was used as a continuous dependent variable without transformation. Model training was implemented using the backpropagation algorithm, with the hyperbolic tangent activation function in the hidden layer and a linear activation function in the output layer. Network parameters were optimized by minimizing the mean squared error (MSE) between observed and predicted fruit weight values.

Model performance and generalization ability were evaluated using 10‐fold cross‐validation. The dataset, comprising 136 genotypes, was randomly divided into 10 folds. In each run, nine folds were used for training and one for validation, generating predictions for all genotypes. Predictive performance was assessed using the coefficient of determination (*R* ^2^), root mean squared error (RMSE), and mean absolute error (MAE). After cross‐validation, the final network was retrained on the entire dataset and subsequently used to estimate the relative importance of each input variable according to the connection‐weight method (Garson 1991; Olden and Jackson 2002). All ANN modeling procedures and performance evaluations were performed in R (Version 4.4.1; R Core Team, Vienna, Austria) using the neuralnet package (Fritsch et al. 2019).

To assess the calibration and generalization performance of the ANN model without information leakage, a simple linear regression was fitted between the observed fruit weight values (*x*) and the corresponding out‐of‐fold predictions (*y*) generated during 10‐fold cross‐validation. Each genotype was predicted only when it belonged to the held‐out test fold and was therefore not used for training the model in that iteration. The resulting calibration equation was *y* = 2.3524 + 0.9511*x*, with *R* ^2^ = 0.9739.

## 3 Results and Discussion

### 3.1 Descriptive Statistics Among Genotypes

Descriptive statistics for the morphological traits evaluated in the examined pear genotypes are presented in Table 1. A one‐way ANOVA was applied to the quantitative traits and revealed statistically significant differences among the genotypes (*p <* 0.05 or *p <* 0.01; Table 1). The highest phenotypic variation was observed in fruit symmetry (207.89%), fruit skin russet (134.42%), fruit skin over color (75.77%), leaf serration depth (69.33%), and fruit breakdown (68.78%). In contrast, the lowest variability was observed for petiole diameter (19.98%), petiole length (19.05%), total soluble solids (18.44%), fruit stalk diameter (18.32%), and leaf length (11.98%). Notably, 35 out of the 40 evaluated traits (87.5%) exhibited coefficients of variation (CV) > 20.00%, indicating a high level of phenotypic diversity among the studied accessions (Khaleghi and Khadivi 2024).

Traits with higher CV values generally provide greater discriminatory power among accessions, genotypes, or cultivars (Khadivi‐Khub and Etemadi‐Khah 2015). Moreover, traits exhibiting wider quantitative ranges often correspond to higher CV values, which further underpins their usefulness for selection in breeding programs (Mohammadi and Prasanna 2003). Conversely, traits characterized by lower CVs reflect more uniform expression across genotypes and thus indicate greater phenotypic stability (Khadivi‐Khub and Etemadi‐Khah 2015). In similar studies, Heidari et al. (2019), Khadivi et al. (2020), and Parvin et al. (2023) reported CV values of 78.26%, 85.40%, and 81%, respectively, and our findings are broadly consistent with previously reported patterns of variation.

Among the quantitative traits listed in Table 1, vegetative leaf traits exhibited substantial variability among the examined genotypes. Leaf length and leaf width ranged from 40.38 (‘Hini‐4’) to 74.78 mm (‘Marivan‐7’) and from 14.57 (‘Kamyaran‐5’) to 48.74 mm (‘Gonjooni‐3’), respectively. In comparison, Heidari et al. (2019) reported narrower ranges for *P. boissieriana* Buhse, with leaf length varying between 19.33 and 51.33 mm and leaf width between 17.13 and 48.75 mm, indicating that the germplasm evaluated in the present study includes genotypes with longer leaves, particularly at the upper end of the distribution. Petiole length and petiole diameter in our material varied from 18.61 (‘Marivan‐4’) to 56.40 mm (‘Dolatabad‐5’) and from 0.39 (‘Gonjooni‐15’) to 1.41 mm (‘Marivan‐12’), respectively, further emphasizing the morphological differentiation in vegetative organs among the studied genotypes. In support of this pattern, Parvin et al. (2023) reported petiole length ranges of 13.30–40.30 mm in *P. glabra* genotypes and 28.60–50.30 mm in *P. syriaca* genotypes, while petiole diameter ranged from 0.70 to 1.14 mm in *P. glabra* and from 0.86 to 1.63 mm in *P. syriaca* . Sharma et al. (2025) also reported petiole length variation in *P. communis* , further corroborating the discriminatory value of petiole‐related traits across *Pyrus* germplasm.

Fruit size‐related traits also showed wide ranges. Fruit length and fruit width varied from 18.80 (‘Khondab‐1’) to 61.10 mm (‘Aghkahriz‐9’) and from 23.80 (‘Bandamir‐3’) to 68.73 mm (‘Kahak‐1’), respectively. Heidari et al. (2019) reported much smaller fruit dimensions for *P. boissieriana* , with fruit length of 8.26–22.51 mm, fruit width of 7.65–24.55 mm, and fruit diameter of 7.36–25.00 mm, whereas Elshihy et al. (2004) documented a fruit length interval of 35.00–120.00 mm in Syrian pear ( *P. syriaca* Boiss.) genotypes. Collectively, these comparisons indicate that the pear genotypes evaluated in the present study occupy an intermediate to high position in terms of fruit size, suggesting apparent interspecific differences between our *P. communis* / *P. syriaca* germplasm and *P. boissieriana* populations. Fruit weight ranged widely from 7.46 (‘Bandamir‐3’) to 139.69 g (‘Kahak‐1’), exceeding the maximum value (93.00 g) reported by Parvin et al. (2025), while Jalilian et al. (2018) reported mean values of fruit length and fruit weight of 7.64 cm and 108.42 g for *P. communis* , and 3.48 cm, 21.41 g for *P. syriaca* . This overall pattern indicates that the current collection includes exceptionally large‐fruited genotypes compared with those previously described in related *Pyrus* species.

Total soluble solids content ranged from 10.00 (‘Dolatabad‐3’) to 26.00% (‘Razghan‐3’), with a mean value of 17.21%. The mean total soluble solids value obtained in the present study (17.21%) is higher than the mean total soluble solids values reported by Jalilian et al. (2018) for *P. communis* (13.07%) and *P. syriaca* (11.30%). In addition, the maximum total soluble solids value recorded in this study (26.00%) exceeds the maximum total soluble solids value of 20.51% reported for *P. glabra* Boiss. and *P. syriaca* Boiss. by Parvin et al. (2025). These findings indicate the presence of genotypes with markedly enhanced sweetness potential, highlighting their considerable importance for both fresh consumption and breeding programs aimed at improving fruit quality.

Internal fruit structure traits also exhibited broad variation: endocarp diameter ranged from 6.70 (‘Bandamir‐13’) to 33.83 mm (‘Dolatabad‐1’), mesocarp diameter from 17.81 (‘Gonjooni‐3’) to 62.52 mm (‘Kahak‐1’), and fruit flesh thickness from 5.33 (‘Marivan‐9’) to 24.92 mm (‘Kahak‐1’). By contrast, Khadivi et al. (2020) reported fruit flesh thickness values ranging from 2.30 to 7.14 mm, suggesting that the genotypes examined here may possess a substantially thicker edible portion than those characterized in some other *Pyrus* germplasm.

Seed‐related traits were likewise variable, with seed number per fruit ranging from 3 (‘Tolaee‐1’, ‘Tolaee‐4’, and ‘Bandamir‐12’) to 15 (‘Kaftarbache‐1’), seed length from 3.50 (‘Aghkahriz‐7’) to 11.53 mm (‘Kamyaran‐4’), seed width from 1.12 (‘Gonjooni‐4’) to 13.26 mm (‘Khondab‐3’), and seed thickness from 0.12 (‘Gonjooni‐4’) to 14.18 mm (‘Aghkahriz‐9’). In *P. boissieriana* , Heidari et al. (2019) reported a seed number of 2–7, seed length of 4.33–8.49 mm, and seed width of 2.48–4.95 mm, values that generally fall within but only partially overlap the ranges obtained in the present study. Overall, the broader intervals and higher upper limits recorded for several key vegetative, fruit, and seed traits reinforce the influence of species identity and germplasm background on phenotypic expression, and underscore the high value of the studied indigenous *P. communis* and *P. syriaca* genotypes for future selection and cultivar development efforts.

### 3.2 Frequency Distribution of Qualitative Morphological Traits

The frequency distribution of qualitative morphological traits demonstrated clear phenotypic structuring among the 136 pear genotypes (Table 2). Oval leaves predominated (122 genotypes), whereas lanceolate leaves were rare (14 genotypes). Similarly, acute leaf bases were observed in 120 genotypes, while rounded bases occurred in only 16, indicating limited variation in basal morphology. Leaf color was predominantly green (91 genotypes), followed by dark green (27) and light green (18), reflecting the prevalence of moderate to high chlorophyll pigmentation. Leaf margin dentation was present in 124 genotypes, with low (86) and intermediate (37) serration depths being the most common, whereas high serration was detected in only one genotype. Serrate margins were the predominant serration type (22 genotypes), while smooth margins were rare (2 genotypes), highlighting the discriminatory value of leaf margin characteristics.

Petiole color displayed substantial variation, with white‐green being the predominant category (68 genotypes), followed by light white (20), yellow‐green (16), white (12), yellow–white (8), yellow (6), yellow–red (5), and white–red (1), indicating its usefulness as a supplementary descriptor for genotype discrimination. Ripening time was predominantly concentrated in the late season, with most genotypes maturing in late September (53), early October (50), and mid‐October (27), whereas only six genotypes ripened between mid‐August and mid‐September. This distribution indicates that the germplasm is enriched in late‐ripening genotypes, offering potential for extending the harvest period. Similarly, Khadivi et al. (2020) reported that most pear genotypes matured from mid‐October to early November, suggesting that the present germplasm represents a relatively earlier ripening spectrum.

Marked variation was observed in fruit traits. Quince‐shaped fruits predominated (119 genotypes), whereas pear‐shaped and apple‐shaped fruits were recorded in only 15 and 2 genotypes, respectively, indicating the prevalence of a single fruit morphotype. In contrast, Khadivi et al. reported pear‐shaped fruits as the dominant type (37 genotypes), with fewer quince‐shaped (14) and apple‐shaped (3) fruits, highlighting population‐specific differences in fruit morphology. Fruit symmetry was absent in most genotypes (110), whereas only 26 were symmetrical, contrasting with the findings of Khadivi et al. (2020), who reported the opposite pattern. Fruit tip shape was relatively balanced between flat (76 genotypes) and round (60 genotypes). Fruit skin ground color exhibited high polymorphism, with light green (39 genotypes), yellow‐green (30), lemon‐yellow (26), green (21), and yellow (20) all well represented. By comparison, fruit skin over color was less variable, being predominantly light green (81 genotypes), followed by lemon‐yellow (32) and yellow (21), while orange‐red over color occurred in only two genotypes, indicating the rarity of strongly blushed fruits.

Fruit skin russet showed considerable variation, being absent in 65 genotypes, low in 55, intermediate in 14, and high in only 4, indicating substantial variation in russet intensity. Fruit skin spot (lenticel) color was predominantly yellow (69 genotypes) or light green (64 genotypes), whereas white (1) and orange‐red (2) were rare, providing additional discriminatory value for fruit skin characteristics. Fruit flesh color was mainly cream (60 genotypes) or white (35 genotypes), while white‐green (24), yellow (9), and white–yellow (8) occurred less frequently, indicating the predominance of light‐colored flesh with appreciable within‐germplasm variation.

Texture‐related traits also exhibited substantial variation. Fruit flesh firmness was predominantly intermediate (60 genotypes) or high (46 genotypes), whereas low firmness was recorded in 30 genotypes, indicating the prevalence of firmer fruits. In contrast, Khadivi et al. (2020) reported lower, intermediate, and higher firmness in 15, 30, and 9 genotypes, respectively, suggesting that the present germplasm contains a greater proportion of firm‐textured fruits. Grit cell amount was mainly low (64 genotypes) or intermediate (58 genotypes), with only 14 genotypes showing high levels, whereas Khadivi et al. (2020) found a higher frequency of highly gritty fruits, indicating a comparatively more favorable texture in the present material. Although grit cell amount was generally low to intermediate, grit cell size was predominantly large (81 genotypes), followed by intermediate (38) and small (17). Similarly, Khadivi et al. (2020) also reported large grit cells as the dominant class, although their germplasm showed a lower proportion of large‐cell phenotypes than observed in the present study.

Organoleptic traits further highlighted the variation of the germplasm. Fruit taste ranged from tasteless (17 genotypes) and sour (4) to sour–sweet (39), slightly sweet (32), sweet (32), and very sweet (12). Similarly, Khadivi et al. (2020) reported broad variation in taste classes, although the higher frequency of sweet and very sweet fruits in the present study indicates a greater proportion of desirable flavor profiles. Overall fruit quality was classified as low in 60 genotypes, intermediate in 42, and high in 34, demonstrating the presence of a considerable number of superior‐quality genotypes. Compared with Khadivi et al. (2020), who reported 19, 23, and 12 genotypes in the low‐, intermediate‐, and high‐quality classes, respectively, the present germplasm contained a relatively higher proportion of high‐quality genotypes. Fruit breakdown was predominantly low (70 genotypes), followed by high (45) and intermediate (21), indicating substantial variation in susceptibility to internal breakdown. Khadivi et al. (2020) similarly reported marked variation for this trait, although the distribution of breakdown classes differed between the two germplasm collections.

**FIGURE 2:**
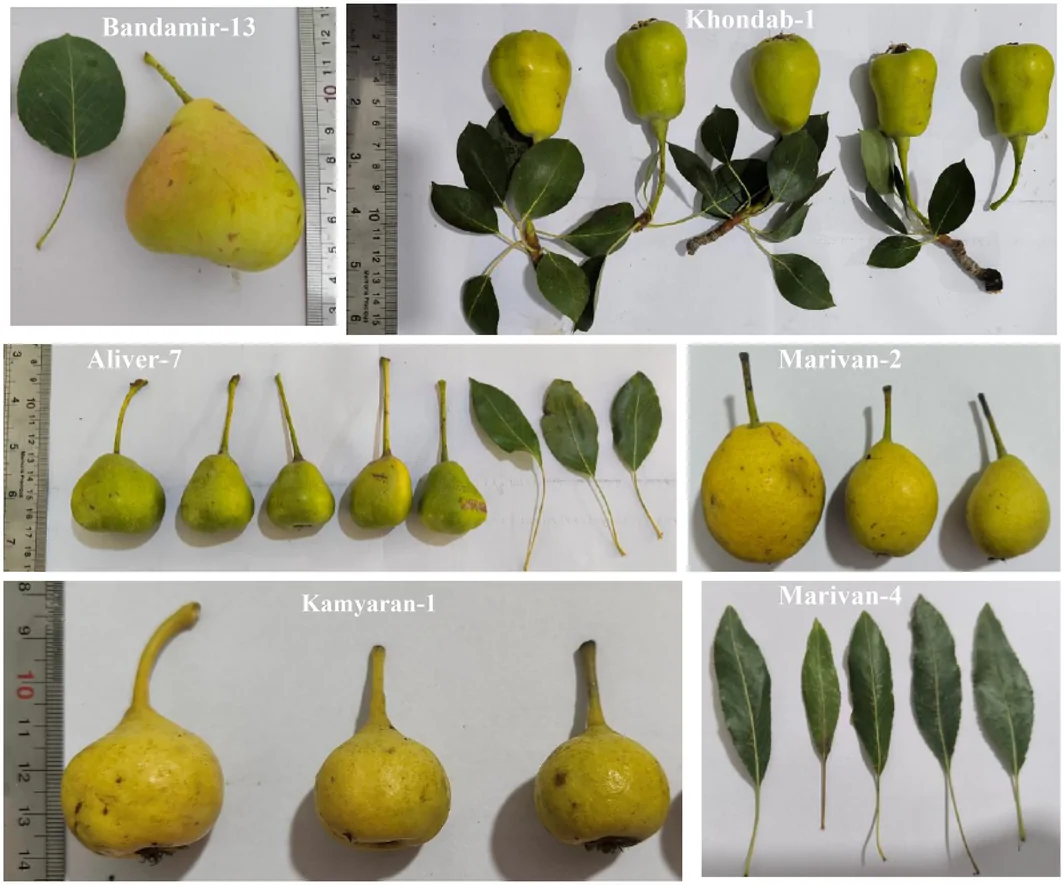
Fruits and leaves of the pear genotypes studied. These images are original and were taken by the first author, Zahra Moradi.

Seed color was relatively evenly distributed among brown (42 genotypes), dark brown (45), and black (49), indicating considerable variation in reproductive pigmentation that may provide supplementary taxonomic value. Overall, comparisons with Khadivi et al. (2020) revealed both similarities and differences in the frequency distributions of key qualitative traits, including ripening time, fruit shape, flesh firmness, taste, fruit quality, fruit breakdown, and grit cell characteristics, highlighting the influence of genetic background and environmental conditions on trait expression in pear. Similarly, Sharma et al. (2025) reported distinct distribution patterns for qualitative fruit traits, supporting the population‐specific nature of these descriptors in *Pyrus*. The variation identified among the studied pear genotypes is presented in Figure 2.

### 3.3 Correlation Analysis

Pearson correlation analysis revealed several biologically meaningful and statistically significant associations among the quantitative traits (Table 3). Fruit weight showed a very strong and positive correlation with both fruit width (*r =* 0.96, *p <* 0.01) and fruit length (*r =* 0.86, *p <* 0.01), indicating that dimensional enlargement is the major predictor of fruit mass in the studied genotypes. Fruit length and fruit width were also highly correlated with each other (*r =* 0.84, *p <* 0.01), reflecting coordinated allometric growth in the longitudinal and transverse axes of the fruit. Together, these strong relationships demonstrate that fruit weight is primarily governed by overall fruit size, and that selection based on fruit dimensions may efficiently improve yield potential.

**TABLE 3:** Pearson correlation coefficients among the evaluated traits in the studied pear genotypes.

Variables	V8	V9	V10	V11	V20	V21	V22	V23	V24	V25	V26	V27	V28	V36	V37	V38	V39
V8	1																
V9	0.24**	1															
V10	0.54**	0.23**	1														
V11	0.23**	0.47**	0.05	1													
V20	−0.09	0.30**	0.18*	0.27**	1												
V21	−0.12	0.15	0.12	0.23**	0.84**	1											
V22	−0.13	0.11	0.10	0.19*	0.86**	0.96**	1										
V23	0.09	0.16	0.06	0.04	0.12	−0.01	0.02	1									
V24	−0.04	−0.19*	−0.07	0.03	0.08	0.29**	0.22*	−0.18*	1								
V25	−0.03	−0.08	−0.10	0.13	0.34**	0.41**	0.41**	−0.01	0.16	1							
V26	−0.18*	0.11	0.06	0.19*	0.78**	0.89**	0.85**	−0.02	0.29**	0.34**	1						
V27	−0.12	0.28**	0.15	0.16	0.77**	0.86**	0.84**	0.01	0.17*	0.09	0.75**	1					
V28	0.05	−0.21*	0.07	−0.27**	−0.35**	−0.39**	−0.37**	0.05	−0.11	−0.19*	−0.34**	−0.33**	1				
V36	−0.03	−0.36**	−0.08	−0.14	−0.06	0.04	0.02	−0.14	0.16	0.35**	0.17*	−0.14	0.05	1			
V37	−0.03	−0.17*	0.02	0.00	0.15	0.23**	0.23**	0.02	0.10	0.45**	0.20*	0.00	−0.19*	0.26**	1		
V38	0.01	−0.38**	−0.03	−0.06	−0.11	0.03	0.08	−0.16	0.09	0.27**	0.00	−0.19*	−0.03	0.20*	0.65**	1	
V39	−0.07	−0.30**	0.02	−0.24**	0.00	0.00	0.06	−0.09	0.02	0.25**	0.00	−0.14	0.09	0.16	0.55**	0.65**	1

*Note:* Abbreviations of traits are provided in Table 1. *, **Correlation is significant at *p* ≤ 0.05 and 0.01 levels, respectively.

Fruit weight exhibited strong and positive correlations with mesocarp diameter (*r =* 0.85, *p <* 0.01) and fruit flesh thickness (*r =* 0.84, *p <* 0.01). These associations indicate that increases in fruit mass are mainly attributable to expansion of the edible portion rather than enlargement of the endocarp. Accordingly, heavier fruits in this germplasm largely correspond to thicker flesh, implying a direct improvement in consumer‐relevant attributes and processing efficiency. The simultaneous strong relationships among fruit weight, mesocarp diameter, and flesh thickness thus highlight these traits as reliable selection criteria in breeding programs aimed at enhancing marketable yield.

Total soluble solids displayed significant negative correlations with both fruit weight (*r =* −0.37, *p <* 0.01) and fruit flesh thickness (*r =* −0.33, *p <* 0.01). These negative associations are consistent with the well‐documented dilution effect, whereby rapid fruit enlargement and increased water accumulation reduce the concentration of soluble sugars on a fresh‐weight basis, even when the absolute amount of sugars continues to increase during fruit development (Dequeker et al. 2024; Zhang et al. 2025). This physiological trade‐off between fruit size and soluble solids has also been associated with fruit water relations and carbohydrate partitioning in pear, indicating that selection exclusively for larger fruits may inadvertently reduce sweetness unless genotypes with favorable sugar accumulation efficiency are identified (Gariglio et al. 2024). Consequently, genotypes deviating from this trend and combining large fruit size with high total soluble solids represent particularly valuable breeding materials.

Seed number per fruit was positively correlated with endocarp diameter (*r =* 0.35, *p <* 0.01) and mesocarp diameter (*r =* 0.17, *p <* 0.05). These relationships suggest that fruits bearing more seeds tend to have larger core and flesh dimensions, consistent with the well‐known hormonal role of developing seeds in stimulating pericarp growth via auxin‐ and gibberellin‐mediated cell division and expansion. This finding emphasizes the developmental linkage between reproductive and pericarp tissues in pear fruit growth.

Among leaf traits, leaf length was positively correlated with leaf width (*r =* 0.24, *p <* 0.01) and petiole length (*r =* 0.54, *p <* 0.01). The latter relationship indicates structural coordination between lamina size and supporting petiole length, reflecting both mechanical support requirements and hydraulic conductance needs for larger leaves. The weaker correlation between leaf length and leaf width suggests partial independence of lamina elongation and broadening, thereby contributing to morphological variation in leaf shape among genotypes.

In summary, the strongest correlations were detected among fruit dimensional traits and fruit weight, followed by relationships involving flesh thickness, mesocarp diameter, and total soluble solids. These patterns collectively indicate that variation in fruit mass is primarily driven by expansion of the edible mesocarp, whereas sugar concentration exhibits a partial negative association with fruit size. The significant association between seed number and pericarp dimensions further supports the physiological coupling between reproductive development and fruit growth in the studied pear genotypes. The statistically significant correlations observed among leaf, fruit, and seed traits are consistent with previously reported findings in pear (Erfani et al. 2014; Rana et al. 2015; Heidari et al. 2019; Khadivi et al. 2020), suggesting that the allometric relationships and developmental linkages identified here represent stable and reproducible patterns across different *Pyrus* germplasm and ecological conditions.

### 3.4 Multiple Regression Analysis (MRA)

In this study, fruit weight, total soluble solids, fruit flesh thickness, and grit cell amount were selected as dependent variables in the multiple regression analysis (Table 4). Fruit weight represents a fundamental quantitative indicator of fruit development and overall pomological performance (Boghean et al. 2025). Total soluble solids content is a key physiological parameter reflecting fruit sweetness and quality (Zheng, Zhang, Wang, et al. 2022). Fruit flesh thickness is a key pomological trait, as it directly reflects the proportion of edible mesocarp and thus strongly influences consumer acceptance, processing suitability, and the overall market value of pears (Mou et al. 2025). Grit cell amount was included due to its critical role in determining flesh texture and eating quality in pear species (Peco et al. 2023).

**TABLE 4:** Characteristics associated with fruit‐related traits in the studied pear genotypes as revealed by MRA.

Dependent character	Independent character	*r*	*r* ^2^	Adjusted *r* ^2^	*β*	*t*‐value	*p*	Sig.	VIF
Fruit weight	Fruit width	0.961^a^	0.92	0.923	0.73	13.83	0.001	**	2.84
	Fruit length	0.967^b^	0.94	0.934	0.25	6.20	0.001	**	1.47
	Seed width	0.970^c^	0.94	0.94	0.07	3.14	0.002	**	3.61
	Fruit tip shape	0.972^d^	0.94	0.943	0.07	3.62	0.001	**	1.25
	Leaf width	0.974^e^	0.95	0.946	−0.09	−3.86	0.001	**	4.19
	Fruit flesh thickness	0.975^f^	0.95	0.948	0.08	2.00	0.048	*	2.05
	Grit cell amount	0.976^g^	0.95	0.949	0.06	2.65	0.009	**	3.12
	Fruit stalk diameter	0.977^h^	0.95	0.951	−0.06	−2.52	0.013	*	1.79
Fruit flesh thickness	Fruit width	0.858^a^	0.74	0.734	0.69	5.43	0.001	**	2.97
	Endocarp diameter	0.906^b^	0.82	0.819	−0.26	−6.83	0.001	**	1.12
	Seed width	0.916^c^	0.84	0.836	−0.12	−3.12	0.002	**	3.44
	Fruit weight	0.920^d^	0.85	0.842	0.30	2.38	0.019	*	2.57
	Leaf width	0.922^e^	0.85	0.845	0.11	2.75	0.007	**	1.66
	Petiole diameter	0.925^f^	0.86	0.849	−0.08	−2.06	0.042	*	2.31
Total soluble solids	Fruit width	0.388^a^	0.15	0.144	−0.30	−4.31	0.001	**	3.82
	Fruit taste	0.525^b^	0.28	0.265	0.26	3.62	0.001	**	1.92
	Ripening date	0.585^c^	0.34	0.327	0.27	4.01	0.001	**	2.69
	Color of spots on fruit skin	0.624^d^	0.39	0.371	−0.23	−3.21	0.002	**	1.33
	Grit cell amount	0.645^e^	0.42	0.394	0.17	2.44	0.016	*	2.18
Grit cell amount	Fruit length	0.424^a^	0.18	0.174	−0.40	−5.46	0.001	**	3.28
	Seed color	0.513^b^	0.26	0.252	−0.26	−3.56	0.001	**	1.58
	Fruit stalk diameter	0.550^c^	0.30	0.287	0.20	2.75	0.007	**	2.44

*Note:* Superscript letters (a–h) indicate stepwise inclusion order of predictors in the MRA models.

Abbreviations: *β*, standardized beta coefficients; *r*, correlation coefficient; *r* ^2^, coefficient of determination; Sig., significance; VIF, variance inflation factor.

*p < 0.05, **p < 0.01.

For fruit weight, the most influential predictor was fruit width (*β* = 0.73, *p <* 0.001, VIF = 2.84), indicating that increases in fruit width substantially enhance overall fruit mass and thus represent a primary driver of pomological performance in the studied genotypes. Fruit length also contributed positively to fruit weight, although to a lesser extent (*β* = 0.25, *p <* 0.001, VIF = 1.47), suggesting that both transverse and longitudinal dimensions jointly determine final fruit size. Seed width exerted a minor but still significant positive effect (*β* = 0.07, *p* = 0.002, VIF = 3.61), implying that genotypes producing larger seeds may tend to develop heavier fruits, possibly reflecting coordinated development between seed and pericarp tissues. Fruit tip shape also positively influenced fruit weight (*β* = 0.07, *p <* 0.001, VIF = 1.25), indicating that specific fruit tip morphologies are associated with larger fruits. By contrast, leaf width showed a significant negative association with fruit weight (*β* = −0.09, *p <* 0.001, VIF = 4.19), suggesting a trade‐off between vegetative growth and photoassimilate allocation to fruit tissues. Fruit flesh thickness had a modest yet significant positive effect (*β* = 0.08, *p* = 0.048, VIF = 2.05), consistent with the expectation that genotypes with thicker edible flesh tend to bear heavier fruits. Grit cell amount also contributed positively (*β* = 0.06, *p* = 0.009, VIF = 3.12), suggesting that genotypes with higher stone cell abundance may accumulate more structural biomass. In contrast, fruit stalk diameter had a negative effect on fruit weight (*β* = −0.06, *p* = 0.013, VIF = 1.79), which may indicate that thicker peduncles do not necessarily correspond to heavier fruits and could even reflect an allocation of resources away from the pericarp.

In the model for fruit flesh thickness, fruit width again emerged as the dominant positive predictor (*β* = 0.69, *p <* 0.001, VIF = 2.97), highlighting a strong linkage between overall fruit diameter and the proportion of edible mesocarp. Endocarp diameter showed a significant negative effect on flesh thickness (*β* = −0.26, *p <* 0.001, VIF = 1.12), indicating that genotypes with larger core or stone regions tend to have reduced flesh thickness, and therefore a lower edible proportion. Seed width was also negatively associated with flesh thickness (*β* = −0.12, *p* = 0.002, VIF = 3.44), reinforcing the notion that increased investment in reproductive structures (seeds, endocarp) can occur at the expense of fleshy tissues. By contrast, fruit weight positively influenced flesh thickness (*β* = 0.30, *p* = 0.019, VIF = 2.57), supporting the idea that heavier fruits, in this germplasm set, are characterized by a more substantial edible portion. Leaf width contributed positively to flesh thickness (*β* = 0.11, *p* = 0.007, VIF = 1.66), suggesting that broader leaves, despite their negative association with fruit weight, may enhance assimilate supply sufficient to promote mesocarp development in certain genotypes. Petiole diameter had a small but significant negative effect (*β* = −0.08, *p* = 0.042, VIF = 2.31), which could be related to differences in leaf structural allocation rather than direct assimilate transport capacity.

Total soluble solids, a key indicator of sweetness and internal quality, were shaped by both morphological and organoleptic traits. Fruit width negatively affected total soluble solids (*β* = −0.30, *p <* 0.001, VIF = 3.82), implying that larger fruits may exhibit a dilution effect, with sugars distributed over a greater volume of tissue, thereby reducing concentration. In contrast, fruit taste, an integrative sensory trait, showed a strong positive association with total soluble solids (*β* = 0.26, *p <* 0.001, VIF = 1.92), confirming that genotypes perceived as better‐tasting indeed have higher soluble solids content. Ripening date also positively influenced total soluble solids (*β* = 0.27, *p <* 0.001, VIF = 2.69), indicating that later‐ripening genotypes generally accumulate more soluble solids, likely due to prolonged maturation and sugar accumulation on the tree. The color of spots on fruit skin was negatively associated with total soluble solids (*β* = −0.23, *p* = 0.002, VIF = 1.33), suggesting that particular pigmentation patterns may be linked with lower sugar concentration or different physiological states. Grit cell amount contributed positively to total soluble solids (*β* = 0.17, *p* = 0.016, VIF = 2.18), which may indicate that genotypes with more developed sclerenchymatous tissue also tend to accumulate higher levels of soluble solids, possibly reflecting shared developmental or metabolic pathways.

Finally, grit cell amount itself was most strongly influenced by fruit length, which showed a marked negative effect (*β* = −0.40, *p <* 0.001, VIF = 3.28), indicating that more elongated fruits tend to have fewer grit cells, whereas shorter fruits are more prone to stone cell accumulation. Seed color exhibited a significant negative association with grit cell amount (*β* = −0.26, *p <* 0.001, VIF = 1.58), implying that darker or more intensely colored seeds may be characteristic of genotypes with reduced sclereid formation in the flesh. In contrast, fruit stalk diameter positively affected grit cell amount (*β* = 0.20, *p* = 0.007, VIF = 2.44), suggesting that genotypes with thicker peduncles may also develop a higher density of stone cells, possibly reflecting a more robust structural phenotype. Overall, the variance inflation factor (VIF) values across all models remained within acceptable limits, indicating that multicollinearity among predictors was not severe and that the estimated standardized coefficients reliably capture the relative contribution of each trait to the studied fruit quality parameters. In the present study, all VIF values were below 5.0, which is commonly considered an acceptable threshold indicating moderate or negligible multicollinearity; values between 5 and 10 are generally interpreted as indicating potentially problematic collinearity, whereas VIF values greater than 10 reflect serious multicollinearity requiring model respecification (Kutner et al. 2005; Hair et al. 2010; Montgomery et al. 2021).

### 3.5 Principal Component Analysis (PCA)

Principal component analysis revealed that the first three principal components explained a substantial proportion of the total phenotypic variation among the studied pear genotypes and captured the major structure of covariation among pomological and morphological characters (Table 5), in line with previous multivariate studies on *Pyrus* germplasm (Zarei et al. 2019; Kadkhodaei et al. 2021; Çoban et al. 2024; Kırca and Kırca 2025). PC1 had an eigenvalue of 6.28 and accounted for 32.33% of the total variance. This component was strongly and positively associated with traits related to fruit size and mesocarp development. The highest loadings were recorded for fruit width (0.96), fruit weight (0.95), mesocarp diameter (0.89), fruit flesh thickness (0.89), and fruit length (0.88), with a moderate contribution of leaf shape (0.53). These patterns clearly indicate that PC1 represented a fruit size–mesocarp expansion axis, on which genotypes with higher scores are characterized by larger, heavier fruits with thicker flesh, whereas those with lower scores exhibited smaller fruit size and reduced mesocarp development. The simultaneous high contributions of fruit width, length, and weight also reinforce the strong functional linkage between longitudinal–transversal fruit growth and overall fruit mass, as previously emphasized in European pear quality assessments (Najafzadeh and Arzani 2015; Zheng, Zhang, Fang, et al. 2022).

**TABLE 5:** Eigenvalues of the principal component axes from the PCA of morphological characters in the studied pear genotypes.

Eigenvectors	Component											
	1	2	3	4	5	6	7	8	9	10	11	12
Leaf shape	0.53^a^	−0.36	0.08	−0.09	0.01	0.24	0.16	−0.41	−0.07	−0.19	0.07	0.12
Leaf shape base	−0.11	−0.21	−0.12	−0.10	−0.03	−0.23	0.67^a^	−0.29	−0.04	0.13	−0.16	−0.14
Leaf color	0.26	−0.11	−0.43	−0.14	−0.15	0.08	0.49	0.19	−0.02	−0.27	0.04	−0.07
Leaf margin dentation (Presence of teeth)	0.07	0.07	0.88^a^	−0.12	0.01	−0.23	−0.09	0.11	−0.01	−0.02	−0.07	−0.04
Leaf serration depth	−0.13	0.00	0.64^a^	0.14	−0.01	−0.03	0.13	−0.02	0.12	0.08	0.40	0.13
Leaf serration shape	0.09	0.11	0.87^a^	−0.13	0.00	−0.22	−0.13	0.16	−0.01	−0.03	−0.01	−0.04
Petiole color	−0.03	−0.23	0.22	0.02	0.31	0.24	0.09	0.54^a^	−0.04	−0.04	0.04	−0.02
Leaf length	−0.18	−0.04	0.00	−0.08	−0.02	−0.01	0.15	0.09	0.86^a^	0.03	0.14	−0.01
Leaf width	0.20	−0.32	−0.18	−0.14	−0.08	0.14	0.54^a^	−0.52	0.26	−0.08	0.03	−0.07
Petiole length	0.14	0.02	0.05	−0.01	0.05	0.08	−0.03	−0.03	0.85^a^	−0.10	−0.16	−0.02
Petiole diameter	0.17	−0.03	−0.05	−0.07	0.06	0.10	0.78^a^	0.09	0.10	−0.07	0.10	0.01
Ripening date	−0.02	0.02	0.53^a^	−0.09	0.07	0.10	−0.19	−0.01	−0.04	−0.03	−0.27	0.47
Fruit shape	0.14	0.16	0.25	0.04	0.09	−0.64^a^	−0.03	0.02	0.01	0.13	0.17	−0.07
Fruit symmetry	−0.15	0.19	−0.13	0.15	0.05	0.65^a^	0.13	−0.13	0.06	0.12	−0.07	−0.01
Fruit tip shape	−0.03	−0.06	−0.15	0.03	−0.19	0.13	−0.06	−0.33	0.05	0.00	−0.18	0.65^a^
Fruit skin ground color	−0.21	0.02	−0.22	0.67^a^	−0.24	−0.17	−0.01	−0.14	−0.12	0.05	0.23	−0.03
Fruit skin over color	−0.14	−0.10	−0.12	0.72^a^	−0.24	−0.20	0.05	0.06	−0.07	−0.04	−0.01	0.11
Fruit skin russet	0.24	0.34	0.23	0.38	0.19	−0.25	0.33	0.07	0.06	−0.16	−0.27	0.12
Color of spots on fruit skin	0.09	−0.05	0.04	−0.69^a^	0.05	−0.19	0.23	−0.06	−0.05	0.03	0.00	−0.04
Fruit length	0.88^a^	0.02	−0.02	−0.10	0.12	0.12	0.11	−0.07	0.03	−0.17	0.02	−0.04
Fruit width	0.96^a^	0.07	−0.01	−0.06	0.04	−0.11	0.07	0.09	0.01	0.02	0.07	−0.05
Fruit weight	0.95^a^	0.12	0.01	−0.08	0.02	−0.08	0.00	0.05	0.00	−0.01	0.06	0.01
Fruit stalk length	0.07	−0.12	−0.09	−0.11	−0.13	0.71^a^	−0.16	0.04	0.07	−0.25	0.23	0.12
Fruit stalk diameter	0.29	0.00	−0.10	0.02	0.00	−0.10	0.06	0.48	−0.03	0.62^a^	0.04	0.03
Endocarp diameter	0.32	0.39	−0.02	−0.02	0.05	0.02	0.10	0.09	−0.09	0.07	0.59^a^	−0.15
Mesocarp diameter	0.89^a^	0.07	0.01	−0.03	0.15	−0.09	0.02	0.07	−0.05	−0.02	0.06	−0.08
Fruit flesh thickness	0.89^a^	−0.17	0.02	−0.07	0.00	−0.08	0.03	−0.03	0.04	−0.02	−0.13	−0.05
Total soluble solids	−0.33	−0.05	0.36	0.44	0.05	0.24	−0.17	−0.05	0.12	0.21	−0.17	0.11
Fruit flesh color	−0.16	0.04	0.18	0.10	0.13	−0.01	−0.03	0.22	−0.07	−0.01	0.14	0.68^a^
Fruit flesh firmness	−0.07	0.07	−0.05	−0.38	0.48	−0.24	−0.04	0.43	0.20	0.03	0.00	0.06
Grit cell amount	−0.32	0.02	0.08	−0.03	−0.02	−0.06	−0.09	−0.05	−0.03	0.77^a^	0.10	0.00
Grit cell size	0.16	0.34	0.13	−0.10	−0.14	−0.21	−0.10	0.57^a^	0.19	0.02	0.11	−0.01
Fruit taste	0.16	0.06	0.29	0.51	−0.17	0.28	−0.06	−0.17	−0.09	0.07	−0.36	−0.14
Fruit quality	0.33	−0.16	0.05	−0.15	0.79^a^	0.02	0.07	−0.12	0.00	−0.14	0.03	−0.14
Fruit breakdown	−0.08	0.01	0.01	0.27	−0.82^a^	0.09	0.02	−0.12	0.02	−0.06	−0.03	−0.10
Seed number per fruit	−0.01	0.29	0.06	0.06	0.46	−0.10	−0.31	0.09	0.00	0.12	0.49	0.05
Seed length	0.14	0.76^a^	0.08	−0.10	0.11	0.09	−0.02	0.02	0.01	−0.04	0.28	−0.05
Seed width	−0.07	0.89^a^	0.02	0.02	−0.05	−0.10	−0.02	0.14	0.01	0.04	0.02	0.08
Seed thickness	−0.02	0.80^a^	0.07	0.05	−0.10	0.01	−0.18	−0.05	−0.02	0.04	−0.02	−0.02
Seed color	0.09	−0.40	−0.02	0.02	0.05	0.21	0.16	0.07	0.28	−0.44	0.13	0.33
Eigenvalue	6.28	4.98	3.10	2.85	2.19	1.95	1.67	1.39	1.27	1.24	1.10	1.07
Component degree of significance	**	**	**	**	**	**	**	*	*	*	*	*
Variance (%)	32.33	20.57	16.49	5.49	3.96	3.86	3.48	3.01	2.94	2.79	1.55	1.39
∑ variance (%)	32.33	52.90	69.39	74.88	78.84	82.70	86.18	89.19	92.13	94.92	96.47	97.86

*Note:* The level of statistical significance of component loadings is indicated as *p < 0.05 and **p < 0.01.

^a^ Component loadings ≥ |0.53| were considered significant.

PC2 had an eigenvalue of 4.98 and explained 20.57% of the variance. In contrast to PC1, this component was primarily defined by seed morphological attributes, including seed width (0.89), seed thickness (0.80), and seed length (0.76). The concentration of high positive loadings on these three traits indicates that PC2 represents a seed size axis, independent from the fruit‐size variation summarized by PC1. Genotypes with high PC2 scores therefore possess larger and thicker seeds, whereas lower scores correspond to smaller‐seeded genotypes. The decoupling between fruit metrics (PC1) and seed metrics (PC2) suggests that seed morphology follows a partly independent developmental trajectory relative to fruit growth, which is consistent with broader evidence on seed–fruit relationships and allometric scaling in fleshy‐fruited species (Ramírez et al. 2021; Rehling et al. 2021; Rojas et al. 2022).

PC3 had an eigenvalue of 3.10 and accounted for 16.49% of the total variance. This component was mainly defined by leaf marginal traits and phenology variables, showing high loadings for leaf margin dentation (presence of teeth) (0.88), leaf serration shape (0.87), and leaf serration depth (0.64), along with a moderate positive loading for ripening date (0.53). Accordingly, PC3 may be interpreted as a leaf serration–ripening time axis. The joint contribution of serration traits and ripening phenology suggests that genotypes exhibiting more pronounced leaf dentation tend to be associated with relatively later ripening behavior, pointing to a potential coordination between vegetative morphology and reproductive timing. Similar linkages between leaf phenology, canopy traits, and seasonal reproductive patterns have been reported for other woody species, highlighting the integrative role of leaf development in determining reproductive schedules under different climatic regimes (Polgar and Primack 2011; Liu et al. 2023; Datta et al. 2025). Although causal relationships cannot be inferred directly from PCA, this pattern highlights a promising subject for further eco‐physiological and genetic investigation.

From a practical standpoint, these findings provide clear guidance for breeding and selection strategies. Large, heavy fruits with thick flesh are clearly associated with high PC1 scores and may be prioritized for fresh‐market quality and consumer preference, as also suggested by previous evaluations of local and commercial pear cultivars (Najafzadeh and Arzani 2015; Akagić et al. 2022). PC2 allows discrimination of genotypes based on seed size, which is relevant for both propagation characteristics and consumer acceptance in cultivars where seed prominence is undesirable. Finally, the PC3 axis can assist in identifying ideotypes combining desired leaf morphology with specific harvest times, enabling the planning of extended harvest seasons and improved adaptation to local growing environments, in agreement with the central role of leaf and reproductive phenology in determining the suitability of fruit trees to particular production regions (Polgar and Primack 2011; Liu et al. 2023).

**FIGURE 3:**
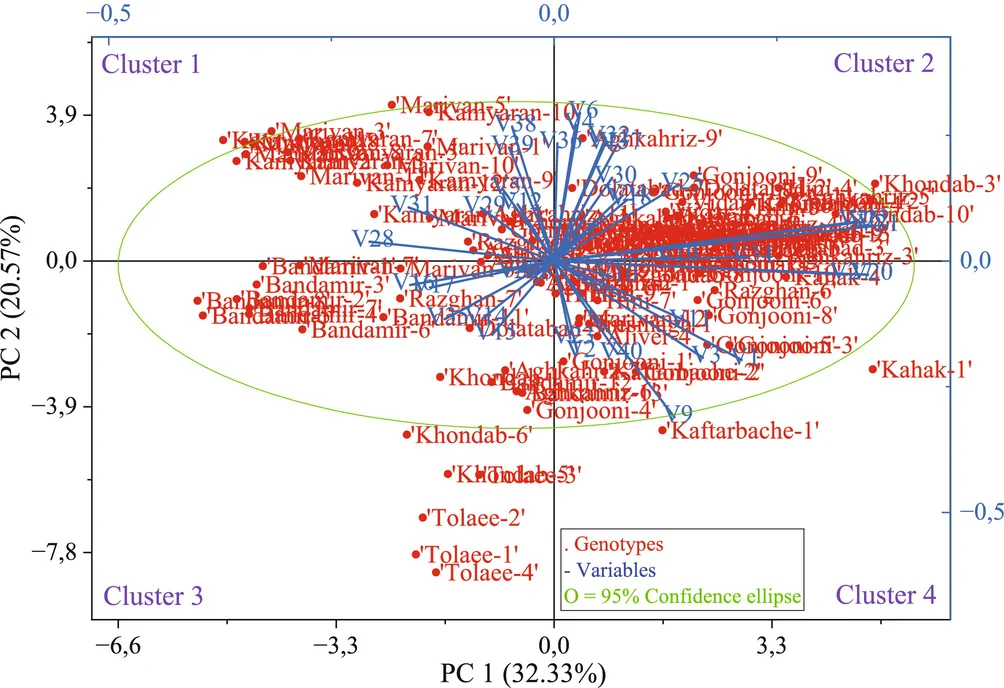
Biplot showing the distribution of the studied pear genotypes and the associated traits based on the first two principal components (PC1 and PC2), which together explained 52.90% of the total variation (PC1 = 32.33%, PC2 = 20.57%). Trait abbreviations are provided in Table 1.

In the PCA biplot, the studied pear genotypes and associated traits were widely dispersed across the four quadrants (Figure 3), indicating a strong and structured multivariate differentiation rather than a random distribution of phenotypes (Abdi and Williams 2010). The 95% confidence ellipse effectively delineated the core multivariate space occupied by the majority of genotypes and thus can be interpreted as the region representing the “average” phenotypic profile of the population (Lê et al. 2008). Genotypes located outside this ellipse are, by definition, multivariate outliers, exhibiting extreme combinations of trait values along the main principal component axes. In the present study, ‘Tolaee‐1’, ‘Tolaee‐2’, ‘Tolaee‐3’, ‘Tolaee‐4’, ‘Kaftarbache‐1’, ‘Kahak‐1’, ‘Khondab‐3’, ‘Khondab‐5’, ‘Khondab‐6’, ‘Kamyaran‐1’, ‘Kamyaran‐8’, ‘Marivan‐3’ and ‘Marivan‐5’ were all positioned beyond the 95% confidence ellipse in the PC1–PC2 biplot, suggesting that they represent structurally distinct phenotypes in the multidimensional trait space (Yan and Kang 2002).

The displacement of these genotypes away from the multivariate centroid is not merely a statistical artifact but reflects biologically meaningful divergence in key pomological and morphological attributes, particularly those related to fruit size, mesocarp development, seed morphology, and, in some cases, flesh texture and internal quality (Escribano et al. 2016). Their extreme scores along the fruit size–mesocarp axis (PC1) and/or the seed size axis (PC2), together with their superior performance for traits such as fruit weight, fruit flesh thickness, and total soluble solids, indicate that these genotypes occupy the upper tail of the phenotypic distribution and therefore constitute valuable promising material. From a genetic resources and breeding perspective, the fact that ‘Tolaee’, ‘Kamyaran’, ‘Khondab’, ‘Marivan’, and ‘Kahak’ genotypes consistently fall outside the 95% confidence ellipse suggests that they harbor unique and potentially complementary allelic combinations that are not captured by the bulk of the collection. Consequently, these genotypes should be prioritized for further evaluation, conservation, and clonal propagation, as they provide excellent candidates for direct cultivar selection, for use as parents in hybridization programs aimed at improving fruit size and quality, and for inclusion in core collections designed to maximize the retention of multivariate phenotypic variation within *Pyrus* germplasm (Volis 2016).

Principal component score analysis (PCSA) was performed to characterize the multivariate variation among pear genotypes based on morphological traits. In the final model, 9 principal components were obtained. Principal component scores and composite scores were calculated for all 136 genotypes, and their values are presented in Table 6. Only the first nine principal components, explaining the majority of the cumulative variance, were retained for the PCSA, whereas the remaining components contributed negligibly and were therefore excluded.

**TABLE 6:** Principal component scores of pear genotypes.

Genotype	PC1	PC2	PC3	PC4	PC5	PC6	PC7	PC8	PC9	Composite score
‘Kamyaran‐1’	5.82	0.70	0.46	0.03	−0.32	0.35	0.20	0.45	0.32	8.01
‘Kahak‐1’	4.91	0.06	0.33	0.79	−0.62	1.07	−0.98	−0.08	0.29	5.77
‘Marivan‐3’	4.36	0.15	−0.38	−0.03	0.16	0.16	0.21	0.81	0.23	5.67
‘Razghan‐3’	5.27	−0.27	0.20	−0.02	0.55	0.06	0.08	−0.18	−0.03	5.66
‘Khondab‐5’	4.00	0.35	0.12	−0.28	−0.64	0.44	0.33	−0.05	0.92	5.19
‘Khondab‐6’	5.64	−0.76	−0.35	−0.02	0.12	−0.12	0.18	−0.63	0.72	4.78
‘Tolaee‐2’	4.18	−0.03	−0.14	0.12	−0.02	−0.14	−0.28	−0.51	1.16	4.34
‘Khondab‐3’	4.55	0.10	0.54	−0.51	0.07	−0.35	0.60	−0.76	−0.28	3.96
‘Tolaee‐3’	5.45	−0.71	0.73	−0.11	0.03	−0.71	−0.27	0.06	−0.58	3.89
‘Kamyaran‐8’	4.73	−0.40	−0.18	−0.20	−0.65	0.58	−0.23	0.17	−0.02	3.80
‘Marivan‐5’	6.00	−0.09	0.01	−0.55	−0.72	0.80	−0.42	−0.50	−1.08	3.45
‘Kaftarbache‐1’	5.09	−0.98	−0.65	0.11	0.37	0.09	−0.06	−0.15	−0.74	3.08
‘Hini‐3’	0.26	0.26	0.75	0.29	0.57	0.44	0.28	−0.16	0.38	3.07
‘Dolatabad‐3’	0.17	−0.37	0.78	0.06	0.59	0.03	1.03	0.88	−0.12	3.05
‘Bandamir‐5’	0.53	−0.26	0.70	1.15	−0.18	−0.22	0.73	0.79	−0.26	2.98
‘Marivan‐2’	1.20	−0.03	0.10	0.01	0.55	0.59	0.32	−0.57	0.80	2.97
‘Bandamir‐12’	−0.24	0.52	0.34	0.92	0.29	−0.18	0.30	0.55	0.41	2.91
‘Bandamir‐13’	0.25	0.53	0.58	0.69	0.32	−0.08	0.07	0.60	−0.41	2.55
‘Khondab‐7’	−0.04	0.56	0.17	0.23	0.28	0.22	0.32	0.66	0.10	2.50
‘Khondab‐2’	1.08	−0.15	0.11	0.12	0.79	−0.05	0.14	0.30	0.09	2.43
‘Aghkahriz‐4’	0.24	0.94	0.67	0.80	−0.26	−0.49	−0.06	0.03	0.55	2.42
‘Kahak‐4’	0.64	−0.30	0.27	−0.10	−0.11	0.55	0.41	0.41	0.65	2.42
‘Aghkahriz‐7’	−0.12	0.35	0.92	0.56	−0.13	−0.55	1.29	0.03	0.01	2.36
‘Kamyaran‐2’	0.52	−0.27	0.66	0.10	1.04	−0.34	0.87	0.10	−0.33	2.35
‘Vesmagh‐7’	−0.39	0.01	0.25	0.73	0.48	1.08	−0.38	0.44	0.09	2.31
‘Gonjooni‐14’	−0.41	−0.04	0.17	0.14	0.41	0.01	0.73	−0.13	1.36	2.24
‘Razghan‐9’	−0.35	−0.02	0.89	−0.31	0.91	0.35	−0.28	0.32	0.49	2.00
‘Tolaee‐1’	0.25	−0.07	0.32	0.76	−0.12	−0.12	0.79	0.38	−0.23	1.96
‘Bandamir‐11’	−0.37	−0.02	0.90	−0.26	0.11	−0.01	0.59	1.26	−0.27	1.93
‘Kamyaran‐3’	−0.24	−0.16	0.21	0.26	−0.29	−0.01	1.07	0.86	0.22	1.92
‘Vidar‐6’	0.12	0.39	−0.74	0.57	0.17	−0.21	0.32	1.14	0.09	1.85
‘Razghan‐8’	0.25	0.09	−0.43	0.35	−0.29	0.06	1.28	−0.05	0.57	1.83
‘Gonjooni‐2’	−0.60	0.41	0.68	−0.04	0.50	0.18	−0.32	0.18	0.77	1.76
‘Gonjooni‐8’	−0.58	0.57	0.38	0.40	−0.45	0.70	−0.70	0.29	1.10	1.71
‘Vesmagh‐9’	0.94	0.48	−0.29	−0.45	0.25	−0.66	0.92	0.59	−0.23	1.55
‘Aliver‐4’	−0.32	−0.54	0.84	0.44	0.00	0.74	0.04	−0.43	0.76	1.53
‘Vesmagh‐6’	0.26	0.77	−0.05	0.20	0.35	−0.20	0.11	0.01	0.05	1.50
‘Kamyaran‐10’	−0.17	−0.17	−0.16	1.04	0.19	0.22	0.52	0.12	−0.13	1.46
‘Kamyaran‐6’	−0.09	0.44	0.33	−0.79	0.74	0.69	−0.31	0.20	0.25	1.46
‘Marivan‐4’	−0.12	0.48	0.59	−0.61	0.30	0.35	−0.15	0.69	−0.08	1.45
‘Marivan‐7’	0.15	−0.69	−0.82	0.51	1.22	0.01	0.28	0.30	0.43	1.39
‘Marivan‐11’	0.28	0.11	0.68	0.06	−0.21	0.06	0.27	0.02	0.02	1.29
‘Gonjooni‐13’	0.58	−0.41	0.48	0.21	0.41	0.95	−0.12	−0.38	−0.44	1.28
‘Vesmagh‐1’	0.01	0.34	−0.16	0.16	−0.07	0.05	0.30	−0.41	1.05	1.27
‘Aghkahriz‐3’	0.14	−0.56	1.22	0.06	0.05	0.36	0.24	0.11	−0.40	1.22
‘Bandamir‐1’	0.31	0.28	0.54	0.42	0.23	−0.04	−0.83	0.21	0.10	1.22
‘Kamyaran‐5’	0.79	−0.61	−0.73	0.11	0.52	0.84	−0.23	0.54	−0.02	1.21
‘Bandamir‐8’	0.14	1.23	−0.32	−0.27	−0.31	−0.28	−0.32	0.59	0.71	1.17
‘Marivan‐13’	0.62	−0.34	0.14	−0.42	1.07	−0.59	0.15	0.32	0.21	1.16
‘Marivan‐1’	−0.53	−0.31	0.96	−0.10	0.11	0.44	0.25	0.08	0.18	1.08
‘Hini‐8’	1.06	0.52	−0.76	−0.24	0.63	−0.35	0.22	0.39	−0.46	1.01
‘Kamyaran‐12’	0.46	0.17	0.50	−1.45	1.04	−0.07	0.55	−0.52	0.31	0.99
‘Aghkahriz‐2’	0.68	0.21	0.94	−0.39	−0.62	−0.89	0.75	0.33	−0.03	0.98
‘Kahak‐3’	0.15	0.41	0.31	−0.41	−0.28	0.37	0.31	−0.01	0.06	0.91
‘Bandamir‐4’	−0.09	−0.22	0.72	0.10	0.52	−0.74	0.13	0.44	0.04	0.90
‘Vidar‐4’	0.20	−0.21	0.14	1.04	0.44	−0.16	0.60	−0.20	−1.02	0.83
‘Aghkahriz‐10’	−0.39	−0.56	0.88	0.47	0.64	0.36	−0.56	−0.26	0.24	0.82
‘Khondab‐1’	0.18	−0.20	0.01	0.64	0.10	0.02	−0.68	0.37	0.32	0.76
‘Gonjooni‐12’	0.12	0.15	−0.36	0.93	0.24	−0.60	0.33	−0.49	0.39	0.71
‘Aliver‐1’	−0.60	0.98	0.02	−0.35	0.11	−0.06	−0.11	0.31	0.38	0.68
‘Gonjooni‐4’	0.18	0.74	−0.26	−0.40	−0.25	0.46	0.16	−0.26	0.26	0.63
‘Razghan‐1’	0.31	0.30	−0.15	0.16	−0.63	0.46	−0.09	−0.26	0.52	0.62
‘Bandamir‐9’	−0.29	−0.42	0.24	−0.28	0.32	0.10	−0.76	0.77	0.90	0.58
‘Khondab‐8’	0.35	−0.04	0.72	−0.34	0.90	−0.02	−0.72	0.06	−0.34	0.57
‘Dolatabad‐5’	−0.13	0.36	0.75	0.04	0.81	−0.69	−0.85	−0.03	0.19	0.45
‘Gonjooni‐7’	0.13	−0.04	−0.96	−0.01	0.03	1.23	−0.10	0.15	−0.02	0.41
‘Hini‐6’	1.07	0.32	−1.01	0.09	−0.33	0.43	−0.40	−0.06	0.25	0.36
‘Bandamir‐2’	0.14	−0.64	−0.54	0.53	−0.02	0.34	0.01	0.01	0.47	0.30
‘Khondab‐10’	0.21	−0.48	−0.02	0.00	−0.58	0.75	0.44	−0.11	0.01	0.22
‘Marivan‐6’	−0.32	−0.66	0.82	0.50	−0.34	1.13	0.49	−0.16	−1.25	0.21
‘Gonjooni‐1’	0.52	0.47	−0.42	−0.15	0.17	0.49	−0.24	−0.09	−0.55	0.20
‘Vesmagh‐5’	0.74	0.43	−0.08	−0.01	−0.50	−0.01	−0.14	0.16	−0.41	0.18
‘Bandamir‐7’	−0.50	0.49	−0.11	−0.02	0.34	−0.56	0.19	0.08	0.25	0.16
‘Hini‐10’	0.72	−0.72	0.58	0.01	−0.49	0.23	0.10	−0.30	0.03	0.16
‘Hini‐2’	0.03	−0.57	0.18	0.28	0.54	0.53	−0.69	−0.47	0.26	0.09
‘Kamyaran‐9’	0.24	0.04	−0.64	0.50	−0.25	−0.78	−0.21	0.75	0.43	0.08
‘Vesmagh‐2’	−0.50	−0.61	0.58	0.40	0.31	0.31	−0.01	−0.45	0.04	0.07
‘Gonjooni‐10’	0.77	−0.39	−0.16	0.41	−0.62	0.11	0.65	−0.80	0.09	0.06
‘Hini‐7’	0.43	−0.60	−0.17	−0.24	−0.33	0.88	0.20	−0.63	0.46	0.00
‘Bandamir‐10’	−0.31	−0.19	0.14	0.17	0.33	1.01	−0.09	−0.40	−0.69	−0.03
‘Aghkahriz‐1’	0.52	−0.26	−0.13	−0.49	−0.22	0.19	0.38	−0.46	0.43	−0.04
‘Razghan‐5’	−0.39	0.10	−0.49	0.20	−0.85	0.51	0.24	0.13	0.49	−0.06
‘Aliver‐3’	0.16	1.54	0.56	−0.06	−0.48	−0.80	0.10	−0.38	−0.71	−0.07
‘Kahak‐2’	0.14	−0.31	−0.10	−0.25	−0.29	0.42	0.18	−0.35	0.45	−0.11
‘Aghkahriz‐11’	−0.61	0.36	−0.12	−0.19	0.36	0.22	−0.18	0.58	−0.54	−0.12
‘Vidar‐3’	−0.30	0.04	−0.08	0.58	0.13	0.17	−0.21	−0.24	−0.22	−0.13
‘Khondab‐4’	0.19	0.78	−0.03	−0.28	0.94	−0.72	−1.10	0.22	−0.25	−0.25
‘Dolatabad‐2’	0.31	−0.53	−0.07	0.06	0.26	0.36	−0.56	−0.77	0.64	−0.30
‘Vidar‐7’	0.12	−0.23	−0.42	0.42	−0.43	0.04	−0.24	0.24	0.17	−0.33
‘Tolaee‐4’	0.19	−0.30	−0.15	−0.30	0.93	−0.01	−0.53	0.41	−0.61	−0.37
‘Gonjooni‐11’	0.13	0.39	−0.62	−0.66	0.26	0.15	0.13	0.17	−0.34	−0.39
‘Gonjooni‐15’	0.31	−0.43	−0.54	0.24	−0.11	0.36	0.24	−0.04	−0.42	−0.39
‘Kamyaran‐11’	−0.10	−0.04	−0.02	0.36	0.03	0.37	−0.04	0.04	−1.00	−0.40
‘Vidar‐2’	0.42	−0.56	0.26	0.72	−1.24	−0.40	0.29	−0.10	0.19	−0.42
‘Gonjooni‐6’	−0.12	−0.71	−0.21	−0.17	−0.40	−0.08	0.20	0.94	0.09	−0.46
‘Aliver‐5’	0.27	−0.52	−0.10	−0.44	−0.69	0.46	0.95	−0.70	0.28	−0.49
‘Razghan‐6’	0.83	0.51	−0.92	−0.64	−0.31	0.01	0.26	−0.36	0.09	−0.53
‘Hini‐5’	0.54	0.03	−0.54	−0.36	0.34	−0.37	0.11	0.02	−0.33	−0.56
‘Dolatabad‐4’	0.49	0.32	0.68	−0.48	0.34	0.53	−0.88	−0.59	−1.02	−0.61
‘Gonjooni‐5’	0.05	0.48	−0.35	−0.16	−0.20	−0.73	0.15	0.13	0.00	−0.63
‘Marivan‐9’	−0.55	0.09	0.70	0.46	−0.79	−0.49	0.47	−0.49	−0.11	−0.71
‘Aliver‐7’	−0.45	−0.29	0.38	0.25	−0.49	0.05	0.38	−0.83	0.27	−0.73
‘Bandamir‐3’	−0.26	0.05	−0.23	−0.22	−0.15	0.11	−0.24	0.63	−0.45	−0.76
‘Kamyaran‐4’	0.02	0.06	0.31	−0.51	−0.13	−0.83	0.20	0.32	−0.24	−0.80
‘Kaftarbache‐2’	−0.36	−0.23	0.53	0.17	−0.88	0.16	−0.19	−0.34	0.31	−0.83
‘Vesmagh‐3’	−0.34	0.49	−0.07	−0.41	−0.16	0.21	−0.28	−0.41	0.12	−0.85
‘Kamyaran‐7’	0.13	−0.28	−0.34	−0.01	0.59	0.27	−0.19	0.39	−1.42	−0.86
‘Dolatabad‐1’	−0.86	0.68	−0.06	0.62	−0.80	−0.30	0.00	0.02	−0.23	−0.93
‘Aliver‐2’	−0.27	−0.29	−0.14	−1.15	−0.76	0.68	0.82	−0.12	0.29	−0.94
‘Razghan‐10’	0.31	−0.79	−0.36	−0.12	−0.04	0.31	0.09	−0.67	0.19	−1.08
‘Khondab‐11’	0.10	−1.02	−0.12	−0.34	−0.50	−0.14	0.90	0.32	−0.29	−1.09
‘Razghan‐4’	0.15	−0.86	−0.67	0.37	0.09	−0.09	0.01	0.17	−0.27	−1.10
‘Hini‐1’	−0.76	−0.22	0.43	0.11	−0.62	0.09	0.19	−0.44	0.08	−1.14
‘Aghkahriz‐8’	−0.01	0.10	−0.07	−0.29	−0.27	−0.02	−0.27	−0.36	0.05	−1.14
‘Hini‐4’	−0.39	−0.12	−0.24	0.04	1.16	−0.93	0.34	−0.81	−0.24	−1.19
‘Gonjooni‐3’	−0.02	0.78	−1.31	0.41	0.04	−0.15	0.05	−0.99	−0.11	−1.30
‘Dolatabad‐7’	−0.03	0.48	−0.49	0.25	−0.27	−0.40	−0.05	−0.52	−0.28	−1.31
‘Razghan‐2’	−0.35	−0.70	−0.78	0.30	−0.64	0.88	−1.04	0.85	0.11	−1.37
‘Marivan‐8’	0.38	0.14	0.05	−0.03	−0.38	−0.14	−0.85	−0.05	−0.49	−1.37
‘Vidar‐5’	−0.50	−0.94	−0.18	0.01	0.84	0.16	−0.11	0.41	−1.11	−1.42
‘Bandamir‐6’	−0.21	−0.14	−0.67	−0.46	−0.50	−0.38	−0.02	0.12	0.78	−1.48
‘Vesmagh‐8’	1.09	−0.40	−0.42	−0.30	−1.06	−0.26	−0.38	0.08	0.17	−1.48
‘Marivan‐12’	−0.35	−0.33	−0.70	0.87	−0.62	−0.35	−0.36	0.45	−0.15	−1.54
‘Marivan‐10’	0.28	−0.48	0.05	−0.67	−0.30	0.16	−0.80	0.22	−0.01	−1.55
‘Razghan‐7’	−0.38	−0.31	−0.70	−0.46	−0.68	−0.49	0.53	−0.47	1.32	−1.64
‘Aghkahriz‐9’	−0.13	0.75	−1.33	0.55	0.62	−1.04	−0.17	−0.19	−0.70	−1.64
‘Aliver‐6’	−0.33	−0.24	−0.30	−0.43	0.02	−0.42	0.14	−0.03	−0.12	−1.71
‘Aghkahriz‐5’	−0.85	0.76	−0.08	−0.21	−0.51	−0.83	0.41	0.04	−0.64	−1.91
‘Vesmagh‐4’	0.12	−0.25	−0.24	0.12	−0.72	−0.70	−0.36	−0.11	0.16	−1.98
‘Aliver‐8’	−0.33	0.29	−0.38	−0.90	−0.81	0.02	0.13	−0.45	0.32	−2.11
‘Vidar‐1’	−0.83	−0.03	−0.61	−0.33	0.02	−0.43	−0.19	0.50	−0.29	−2.19
‘Gonjooni‐9’	−0.50	−0.28	0.05	−0.25	−0.78	0.03	−0.53	0.24	−0.46	−2.48
‘Dolatabad‐6’	−0.02	−1.03	−0.04	−0.65	0.33	0.18	−0.47	−0.26	−0.53	−2.49
‘Aghkahriz‐6’	−0.65	−0.17	0.83	−0.13	−0.75	−0.12	−0.14	−1.35	−0.03	−2.51
‘Khondab‐9’	0.42	−0.33	−0.22	−0.94	−0.23	−1.21	−0.79	0.38	0.39	−2.53
‘Hini‐9’	−0.03	−1.62	−0.51	−0.13	−0.62	0.82	−0.72	−0.22	0.07	−2.96

The 12 top‐ranked genotypes were ‘Kamyaran‐1’ (8.01), ‘Kahak‐1’ (5.77), ‘Marivan‐3’ (5.67), ‘Razghan‐3’ (5.66), ‘Khondab‐5’ (5.19), ‘Khondab‐6’ (4.78), ‘Tolaee‐2’ (4.34), ‘Khondab‐3’ (3.96), ‘Tolaee‐3’ (3.89), ‘Kamyaran‐8’ (3.80), ‘Marivan‐5’ (3.45), and ‘Kaftarbache‐1’ (3.08), all of which were positioned outside the 95% confidence ellipse in the PC1–PC2 biplot, indicating that they represent structurally distinct, extreme phenotypes in the multivariate trait space. In contrast, the five lowest‐scoring genotypes were ‘Gonjooni‐9’ (−2.48), ‘Dolatabad‐6’ (−2.49), ‘Aghkahriz‐6’ (−2.51), ‘Khondab‐9’ (−2.53), and ‘Hini‐9’ (−2.96), occupying the opposite extreme of the principal component analysis ordination and reflecting inferior overall performance from a multivariate perspective. Among the promising group, ‘Kahak‐1’ had the highest fruit weight (139.69 g) and the greatest fruit flesh thickness (24.92 mm), while ‘Razghan‐3’ exhibited the highest total soluble solids content (26%), underscoring their superior pomological profiles. Furthermore, several genotypes, including ‘Gonjooni‐4’, ‘Gonjooni‐7’, ‘Gonjooni‐14’, ‘Gonjooni‐15’, ‘Bandamir‐11’, ‘Kamyaran‐1’, ‘Kamyaran‐5’, ‘Kamyaran‐7’, ‘Kamyaran‐8’, ‘Kamyaran‐12’, ‘Marivan‐1’, ‘Marivan‐4’, and ‘Marivan‐7’, displayed high scores for grit cell amount; notably, ‘Kamyaran‐1’, which also lay outside the 95% confidence ellipse, linked its extreme principal component scores to a distinct flesh texture pattern. Taken together, these findings demonstrate that the principal component scores‐based ranking is strongly supported by the descriptive statistics, principal component analysis loadings, biplot configuration, and multiple regression analysis, and therefore reliably captures the major sources of phenotypic divergence among the studied pear genotypes.

This approach offers a novel framework for ranking pear genotypes based on multi‐trait composite scores. This independent evaluation allows the inferences and conclusions derived from our dataset to be both meaningful and inherently original, providing new insights into morphological variation without being limited by the absence of directly comparable multivariate assessments in earlier research.

### 3.6 Hierarchical Cluster Analysis (HCA)

**FIGURE 4:**
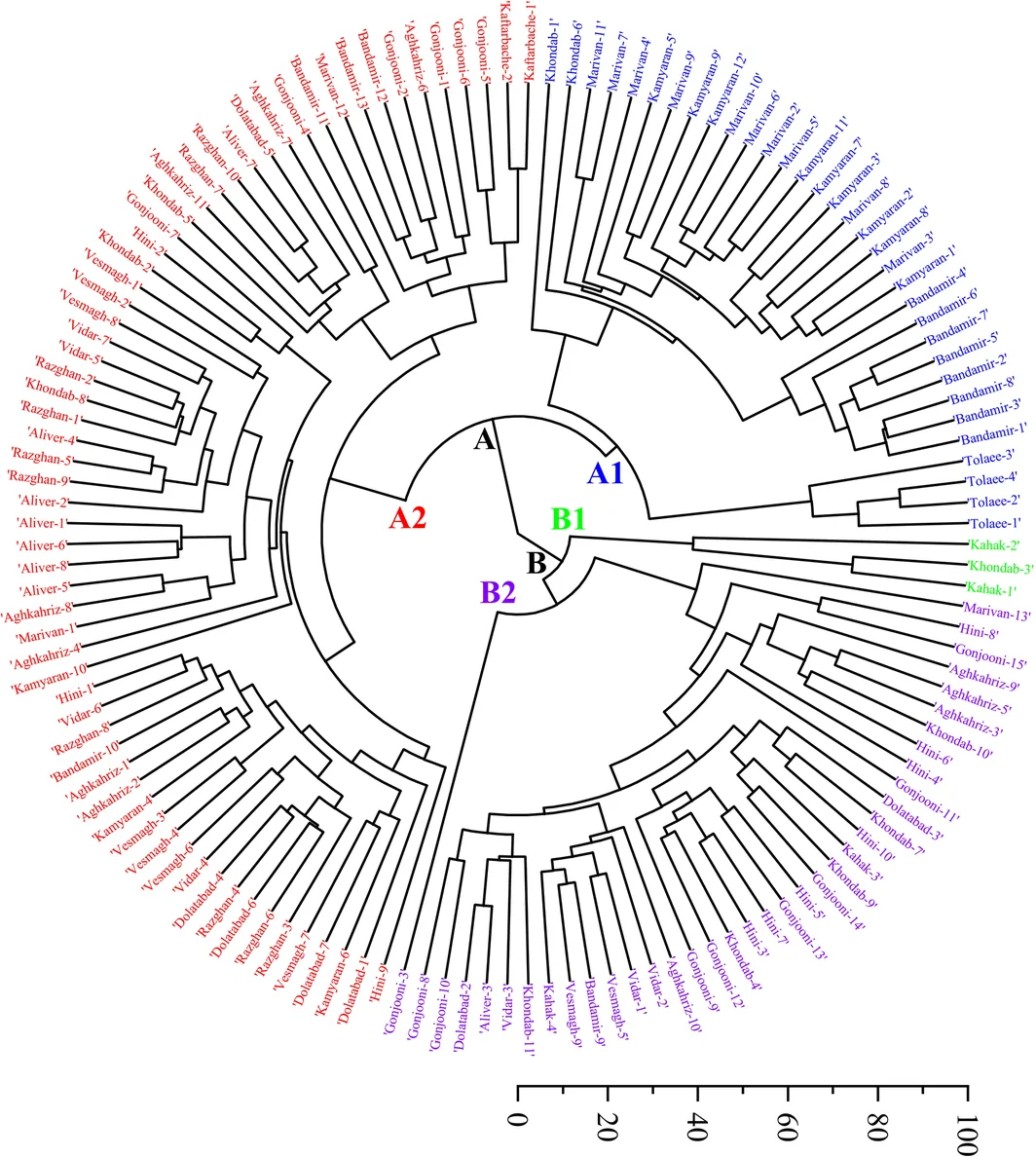
Hierarchical cluster analysis‐based visualization of morphological clustering patterns among pear genotypes.

The clustering patterns of pear genotypes derived from morphological traits are illustrated in Figure 4, and the detailed agglomeration steps obtained using Euclidean distance coefficients are presented in Table S2. The hierarchical classification successfully discriminated among the 136 pear genotypes collected from different regions of Kurdistan, Isfahan, and Markazi provinces, revealing the phenotypic structure within the germplasm, in line with recent multivariate studies on pear germplasm, where HCA based on Euclidean metrics has been effectively used to resolve variation patterns (Simionca Mărcășan et al. 2023). The generated dendrogram exhibited a clear hierarchical arrangement in which genotypes were progressively merged from small units into larger clusters, thus representing a coherent grouping pattern. This hierarchical ordering effectively captured the gradient of phenotypic variation, with lower linkage distances reflecting close morphological resemblance and higher linkage distances corresponding to greater divergence among genotypes.

At the earliest stage of clustering, the genotypes ‘Bandamir‐5’ and ‘Bandamir‐7’ were fused at a minimum Euclidean distance of 8.82 (Stage 1), indicating a very close phenotypic affinity and suggesting that these genotypes may have evolved under similar selection pressures. As the clustering process progressed, the linkage distances gradually increased, reaching a maximum value of 65.85 at Stage 135, where the two most divergent genotypes, ‘Tolaee‐1’ and ‘Gonjooni‐3’, were finally merged. The wide range of observed Euclidean distances (8.82–65.85) clearly highlights the pronounced morphological heterogeneity present in the studied population, a pattern comparable to that reported for other woody perennials where Ward‐type HCA with Euclidean distance has been applied to partition highly variable germplasm into homogeneous groups (Maleki‐Meighani et al. 2025).

The existence of both very closely related pairs and highly divergent genotypes suggests that the germplasm pool comprises mixtures of locally adapted forms, potentially clonal lineages, as well as highly distinct phenotypes that may represent unique selections or ecotypes. Such variation is expected in open‐pollinated, long‐lived perennial fruit trees that experience strong environmental heterogeneity and human selection over time, and similar patterns have been documented in wild and cultivated pear complexes across Eurasia (Paganová 2009). The clear group formation observed in the dendrogram further supports the results of PCA and MRA, confirming that fruit dimensional traits, seed characteristics, and leaf morphology exert strong discriminatory power among the evaluated genotypes, consistent with recent reports that identified fruit size, external appearance, and internal quality descriptors as key contributors to phenotypic differentiation in pear germplasm collections (Postman 2019).

From a breeding and conservation perspective, closely clustered genotypes represent related materials, whereas they may provide promising contrasting parents for future crossing programs aimed at broadening phenotypic diversity (Labroo et al. 2021). Conversely, tightly clustered genotypes such as ‘Bandamir‐5’ and ‘Bandamir‐7’ may be of interest for further evaluation in clonal propagation and orchard establishment.

Overall, the HCA results corroborate the high level of phenotypic variation revealed by descriptive statistics, frequency distributions, and PCA, and emphasize the importance of indigenous pear germplasm as a reservoir of functional variation for future breeding, conservation, and domestication initiatives (Kırca and Aygün 2025).

In addition, clustroid analysis was performed to identify the most and least representative genotypes within each cluster based on the sum of distances (Table 7). In Cluster 1, ‘Razghan‐9’ was recognized as the most representative observation (clustroid), whereas ‘Kahak‐1’ was the least representative member, occupying a more peripheral position in the multivariate trait space. These results are helpful in selecting core genotypes that best represent the overall structure of each cluster and for recognizing extreme or atypical phenotypes among the studied pear genotypes. Furthermore, by quantitatively distinguishing central and peripheral members of each cluster, clustroid analysis provides a practical framework for designing core collections and for parent selection in breeding programs, where clustroid genotypes may be regarded as typical representatives of the population and peripheral genotypes as valuable sources of extreme trait expression and broader phenotypic variability, in line with recent studies emphasizing optimized core collections for genetic and pre‐breeding studies (Gouy et al. 2025; Osorio et al. 2025).

**TABLE 7:** Clustroid analysis of representative and peripheral pear genotypes.

Clustroid info		
Cluster	Most representative observation	Least representative observation
1	‘Razghan‐9’	‘Kahak‐1’

*Note:* Method: Sum of distances.

### 3.7 ANN‐Based Fruit Weight Prediction

The ANN model developed for fruit weight prediction demonstrated high predictive performance. Using eight morpho‐pomological predictors (fruit width, fruit length, seed width, fruit tip shape, leaf width, fruit flesh thickness, grit cell amount, and fruit stalk diameter), the MLP with a single hidden layer (5 neurons) achieved a cross‐validated coefficient of determination of *R* ^2^ = 0.9739, with a root mean squared error of 5.34 g and a mean absolute error of 4.11 g. Considering that the observed fruit weight ranged from 7.46 to 139.69 g with a standard deviation of 29.85 g, these error values indicate strong agreement between observed and predicted values and a substantial gain in explanatory power compared with classical linear models.

**FIGURE 5:**
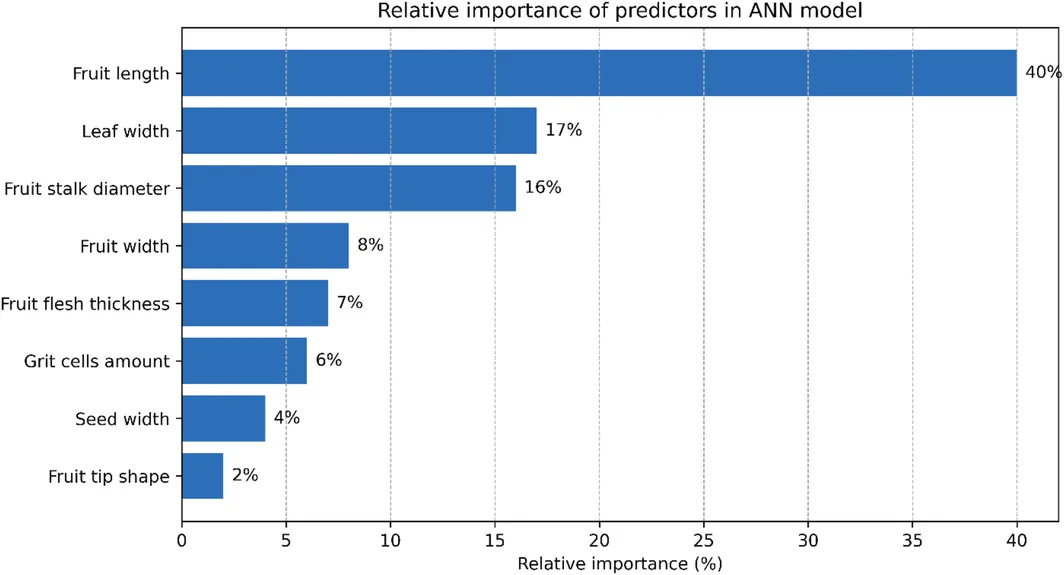
Relative importance of morphological and pomological predictors in the artificial neural network model for fruit weight in 136 pear genotypes. Variable importance was quantified using the connection‐weight method based on the products of input–hidden and hidden–output weights.

The connection‐weight analysis revealed that fruit length was the most influential predictor, accounting for approximately 40% of the total relative importance, followed by leaf width (17%) and fruit stalk diameter (16%). Fruit width contributed about 8%, whereas fruit flesh thickness and grit cell amount accounted for 7% and 6%, respectively. Seed width and fruit tip shape had relatively minor roles, each contributing < 5% to the overall importance (Figure 5). These results collectively highlight the central role of fruit dimensional traits, particularly longitudinal growth (fruit length), in determining fruit weight, and underline the additional contribution of leaf morphology and stalk diameter, which may reflect differences in assimilate supply and vascular support.

Overall, Figure 5 demonstrates that fruit weight in pear is shaped by a complex interplay of morphological traits encompassing size dimensions, photosynthetic source capacity, and vascular transport characteristics. The biologically interpretable hierarchy of variable importance obtained from the ANN model is consistent with previous applications of neural networks in horticultural species, where ANN‐based models often outperform classical linear regression when trait relationships are strongly nonlinear (Abdel‐Sattar et al. 2021; Bharti et al. 2023). These findings emphasize that fruit length, leaf width, and fruit stalk diameter represent key predictors of fruit weight and should be considered as primary selection criteria in pear breeding programs aimed at improving fruit size and yield potential.

Recent work in fruit crops increasingly demonstrates the value of ANN‐based models for predicting yield‐related traits from a limited set of easily measured predictors. In pear, ANN architectures have been successfully used to estimate mechanical properties such as rupture energy in ‘Deveci’ and ‘Abate Fetel’ fruits with high predictive accuracy (*R* ^2^ up to ~0.94), underscoring the capacity of neural networks to capture complex, nonlinear relationships among morpho‐mechanical traits (Cevher and Yıldırım 2022). Similar ANN and deep‐learning frameworks have been developed for predicting fruit yield or single‐fruit weight in other species, including apple, mango, and loquat, using morphological and phenological inputs or image‐derived features, and these studies consistently report superior performance of machine‐learning approaches compared with classical linear models (Huang et al. 2021; Bharti et al. 2023; Alebidi et al. 2025). In parallel, recent work on wild pear germplasm highlights substantial variability in fruit dimensions, seed traits, and internal quality attributes, reinforcing the relevance of morpho‐pomological descriptors as informative predictors in data‐driven models of fruit quality and size (Khadivi et al. 2020; Vrtodusic and Babojelić 2022; Kırca and Aygün 2025).

Similar findings have been reported in recent food and horticultural studies. Sasikumar et al. (2024) successfully predicted the mass of *Elaeagnus latifolia* fruits using ANN, achieving high prediction accuracy (*R* ^2^ = 0.9901), and concluded that ANN provides an efficient alternative to conventional mathematical models for modeling complex fruit characteristics. Likewise, Sasikumar et al. (2023) demonstrated that ANN outperformed traditional mathematical drying models for predicting drying kinetics in red bell pepper, highlighting its superior capability to capture nonlinear biological responses. More recently, Nongmaithem et al. (2026) reported that a hybrid ANN–genetic algorithm (ANN–GA) model exhibited substantially greater predictive accuracy than response surface methodology during the optimization of probiotic Sohiong beverage production, further confirming the robustness of ANN‐based approaches for complex biological systems. Although these studies addressed different agricultural and food‐processing applications, their findings consistently demonstrate that ANN‐based models provide reliable and highly accurate predictions in systems characterized by complex nonlinear interactions. The high predictive accuracy obtained in the present study therefore supports the application of ANN as an effective decision‐support tool for genotype evaluation and breeding programs aimed at improving fruit size and related pomological traits in pear.

**FIGURE 6:**
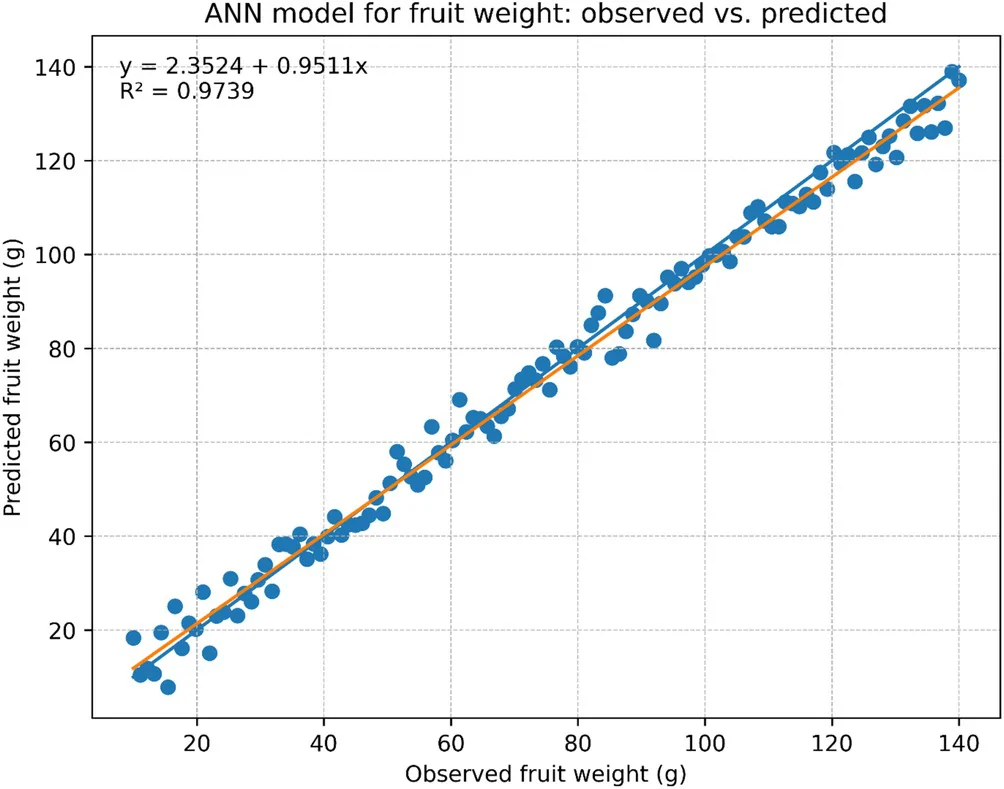
Observed versus out‐of‐fold ANN‐predicted fruit weight values for 136 pear genotypes obtained from 10‐fold cross‐validation. Each predicted value represents a test‐fold prediction generated when the corresponding genotype was excluded from model training. The solid line represents the 1:1 relationship between observed and cross‐validated predicted values.

The ANN model developed in this study showed a very high predictive capability for fruit weight in pear genotypes. The strong agreement between observed and ANN‐predicted values is clearly illustrated in Figure 6, where the data points are tightly clustered around the 1:1 line. The calibration regression fitted between observed and predicted values yielded the equation *y* = 2.3524 + 0.9511*x* with a coefficient of determination of *R* ^2^ = 0.9739, indicating a strong agreement between observed and predicted values. The slope close to unity and the small dispersion of points around the identity line demonstrate both high accuracy and precision of the model.

**FIGURE 7:**
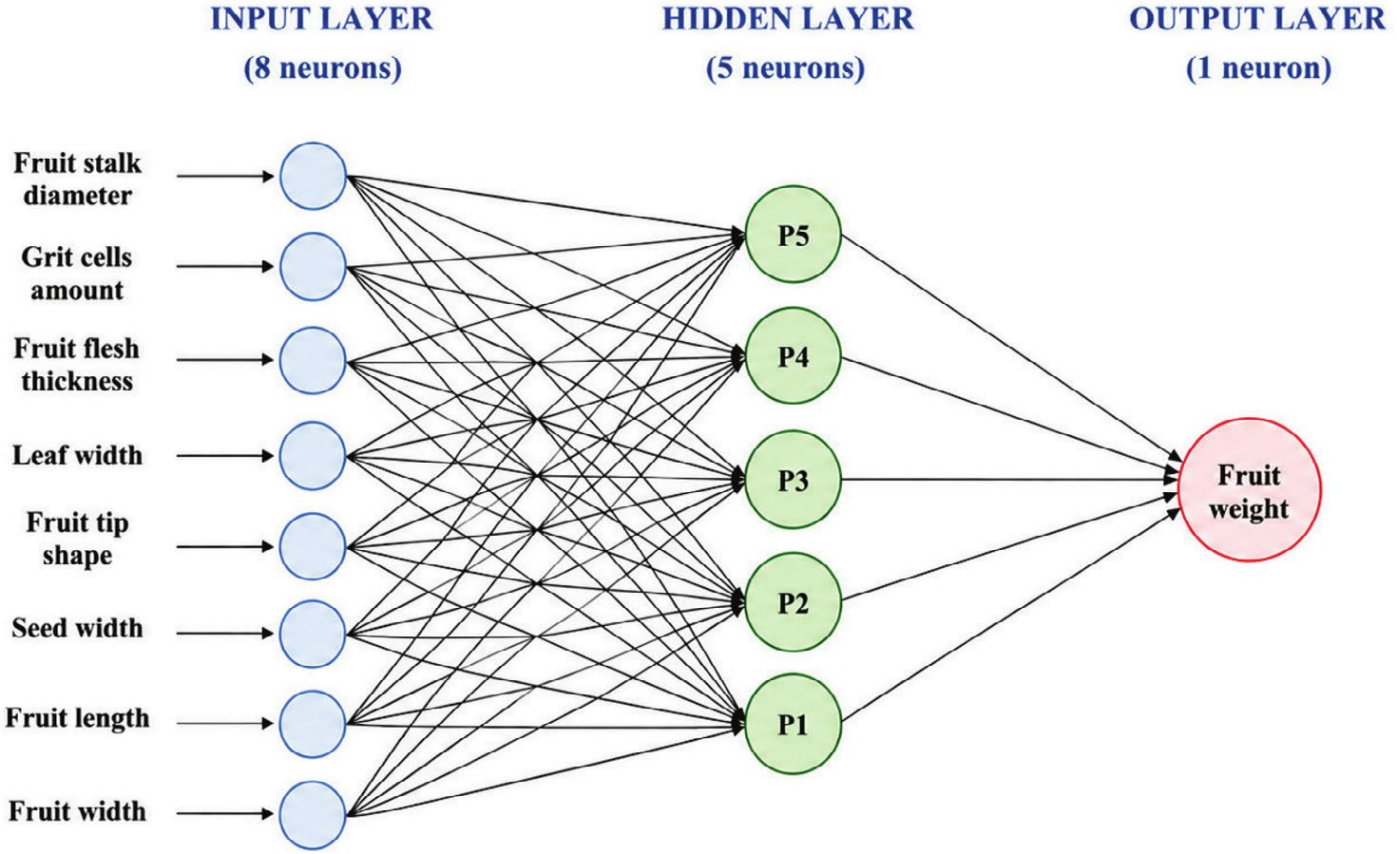
Schematic representation of the feed‐forward multilayer perceptron (MLP) architecture used for fruit weight prediction. The network consists of an input layer with eight morpho‐pomological predictors, one hidden layer with five neurons, and a single output neuron representing predicted fruit weight. All neurons in adjacent layers are fully connected through weighted connections.

Figure 7 illustrates the relative importance of the predictor variables included in the ANN model for fruit weight, revealing a clear hierarchical structure among traits. Fruit length emerged as the most influential variable, indicating that longitudinal expansion is the primary predictor of final fruit weight and a key driver of fruit volume and biomass accumulation. This result is consistent with previous ANN‐based work in fruit crops, where size‐ and geometry‐related attributes are strong predictors of mechanical or yield‐related traits (Ziaratban et al. 2017; Cevher and Yıldırım 2022).

Fruit length was followed by leaf width and fruit stalk diameter, both of which exhibited high relative importance. The strong contribution of leaf width is biologically plausible, as greater leaf area is closely associated with enhanced photosynthetic capacity and thus a larger supply of assimilates to developing fruits. Similar source–sink relationships have been reported in ANN models that predict fruit quality or yield from physiological and nutritional variables, highlighting the role of source strength in determining fruit growth and quality (Huang et al. 2021, 2022). The substantial importance of fruit stalk diameter likely reflects variation in vascular transport efficiency; a thicker peduncle may facilitate more effective transport of water and carbohydrates to the fruit, promoting cell expansion and fruit filling.

Fruit width and fruit flesh thickness played a moderate role in the model, supporting the notion that fruit weight is not governed by a single dimensional parameter but rather by the integrated effect of longitudinal, transversal, and radial growth. The contribution of grit cell amount, although smaller, suggests that anatomical differences in tissue structure and density also influence total fruit mass, in line with studies that show that ANN models have captured subtle textural or structural effects on fruit physical properties (Amoriello et al. 2022; Cevher and Yıldırım 2022). In contrast, seed width and fruit tip shape showed comparatively low relative importance, indicating that their effects on fruit weight are indirect or minor and are more likely associated with morphological variation than with biomass accumulation themselves.

To further evaluate the robustness of the proposed MLP model, its predictive performance should be interpreted in the context of other widely used machine‐learning algorithms. Recent studies have emphasized that comparative evaluation of ANN, support vector regression (SVR), random forest (RF), and conventional regression models is essential because no single algorithm consistently performs best across different agricultural datasets. Instead, prediction accuracy largely depends on dataset characteristics, sample size, predictor interactions, and the degree of nonlinearity among variables (Debnath 2024; Opara et al. 2024; Sharma et al. 2024).

Fruit weight is a quantitative trait governed by multiple highly correlated morpho‐pomological variables, including fruit width, fruit length, mesocarp diameter, and flesh thickness. Such relationships are inherently nonlinear and involve complex interactions that cannot always be adequately represented by conventional linear regression models. Artificial neural networks, particularly multilayer perceptrons (MLPs), are well suited to capture these nonlinear dependencies through adaptive learning of hidden feature representations, thereby improving predictive accuracy in biological datasets (Mancero‐Castillo et al. 2024; Sabouri et al. 2025).

Recent horticultural studies have similarly demonstrated that ANN‐based models frequently achieve superior predictive performance compared with traditional statistical approaches and several conventional machine‐learning algorithms when modeling fruit characteristics and yield‐related traits, although comparative benchmarking remains necessary for each specific dataset. Therefore, evaluating MLP together with algorithms such as SVR, RF, and multiple linear regression provides a more comprehensive assessment of model reliability and generalization performance (Mancero‐Castillo et al. 2024; Opara et al. 2024; Sabouri et al. 2025).

### 3.8 Limitations of the Study

Despite providing a comprehensive morpho‐pomological characterization and high‐performance ANN modeling of indigenous pear genotypes, several limitations should be acknowledged. First, although the evaluations were conducted over two consecutive growing seasons, all genotypes were collected from only three provinces of Iran, which may limit the broader environmental representativeness of the findings. Second, the present study focused exclusively on morphological and pomological descriptors without integrating molecular marker or genomic information that could further validate the observed phenotypic relationships. Third, the ANN model was developed and internally validated using the available dataset; therefore, its predictive performance should be further assessed using independent external populations collected from different ecological regions. Finally, environmental variables such as soil characteristics, climatic conditions, and orchard management practices were not explicitly incorporated into the prediction model, although these factors may influence phenotypic expression. Future studies combining multi‐environment trials, genomic datasets, and external ANN validation are expected to improve the robustness and general applicability of the proposed prediction framework.

## 4 Conclusions

The present study revealed substantial morphological and pomological variation among 136 indigenous *P. communis* and *P. syriaca* genotypes, with most traits exhibiting wide ranges and coefficients of variation > 20%, confirming the rich genetic potential of this germplasm for breeding and domestication. Multivariate analyses (PCA, HCA, and principal component score analysis) revealed that fruit size–mesocarp development, seed morphology and leaf serration–ripening behavior constitute the main axes of phenotypic differentiation, and highlighted a structured separation of genotypes rather than a random distribution in the multivariate trait space.

Based on composite principal component scores, 12 promising genotypes—‘Kamyaran‐1’, ‘Kahak‐1’, ‘Marivan‐3’, ‘Razghan‐3’, ‘Khondab‐5’, ‘Khondab‐6’, ‘Tolaee‐2’, ‘Khondab‐3’, ‘Tolaee‐3’, ‘Kamyaran‐8’, ‘Marivan‐5’, and ‘Kaftarbache‐1’—were identified as top‐ranked genotypes positioned outside the 95% confidence ellipse, indicating structurally distinct and superior phenotypes. Within this group, ‘Kahak‐1’ had the highest fruit weight and flesh thickness, whereas ‘Razghan‐3’ exhibited the highest total soluble solids content, highlighting their particular value as candidates for fresh‐market cultivars and as parents in breeding programs aiming to improve fruit size and internal quality.

The ANN model developed in this study, using eight easily measurable morpho‐pomological predictors, achieved an excellent predictive performance for fruit weight (*R* ^2^ = 0.9739 with low RMSE and MAE values), and connection‐weight analysis showed that fruit length, leaf width, and fruit stalk diameter were the most influential predictors of fruit mass. These findings indicate that the integration of detailed morpho‐pomological characterization with ANN‐based modeling provides a robust, biologically interpretable framework for both the identification of promising genotypes and the formulation of practical selection criteria. The proposed ANN model provides a practical tool for the rapid selection of superior genotypes and supports more efficient germplasm management in pear breeding programs. Nevertheless, although the ANN demonstrated high predictive performance under cross‐validation, the present study was not designed as a comprehensive machine‐learning benchmarking analysis. Future studies using larger independent datasets should compare ANN with alternative algorithms, particularly support vector regression and Random Forest, using harmonized hyperparameter optimization and validation procedures to determine whether the observed predictive performance is model‐specific or consistent across different nonlinear learning approaches. Future work should integrate multi‐year, multi‐environment trials and genomic information to validate model robustness and to enhance the efficiency of genotype selection and conservation strategies.

## Author Contributions

**Zahra Moradi:** conceptualization, investigation, methodology, data curation, resources, writing – review and editing. **Ali Khadivi:** conceptualization, investigation, funding acquisition, writing – original draft, methodology, validation, visualization, supervision, writing – review and editing, project administration. **Yazgan Tunç:** writing – original draft, validation, visualization, software, formal analysis, data curation.

## Funding

The authors have nothing to report.

## Disclosure

Statement specifying permissions: For this study, we acquired permission to study pear issued by the Agricultural and Natural Resources Ministry of Iran.

Statement on experimental research and field studies on plants: The cultivated or wild‐growing plants sampled comply with relevant institutional, national, and international guidelines and domestic legislation of Iran.

## Conflicts of Interest

The authors declare no conflicts of interest.

## Supporting information


**Table S1:** Collection sites and geographic coordinates of the evaluated pear genotypes.
**Table S2:** Cluster formation stages of pear genotypes.

## Data Availability

The data that support the findings of this study are available from the corresponding author upon reasonable request.
