# Supplementary material for: Morphological and Pomological Characterization of Indigenous Pear Genotypes (Pyrus communis L. and P. syriaca Boiss.) and ANN‐Based Prediction of Fruit Weight
Source: Food Sci Nutr. 2026 Sep 21;14(9):e72401. doi: 10.1002/fsn3.72401 (PMC13594562; doi:10.1002/fsn3.72401)
Supplement: Supplementary file 1 [file FSN3-14-e72401-s001.docx]

**S1 Table.** Collection sites and geographic coordinates of the evaluated pear genotypes.

| Province | Region | Area | Number of genotypes (n) | Latitude (N) | Longitude (E) | Altitude (m) |
| --- | --- | --- | --- | --- | --- | --- |
| Kurdistan | Marivan | Lavisan | 4 | 46° 28′ 40″ | 35° 26′ 22″ | 1533 |
| Kurdistan | Marivan | Varo | 4 | 46° 28′ 19″ | 35° 28′ 39″ | 1534 |
| Kurdistan | Marivan | Darzian | 5 | 46° 11′ 09″ | 35° 26′ 34″ | 1370 |
| Kurdistan | Kamiaran | Yamenan | 3 | 46° 57′ 10″ | 35° 01′ 26″ | 1380 |
| Kurdistan | Kamiaran | Naleshken | 3 | 46° 54′ 37″ | 34° 39′ 52″ | 1410 |
| Kurdistan | Kamiaran | Gashki | 3 | 46° 51′ 14″ | 34° 49′ 09″ | 1450 |
| Kurdistan | Kamiaran | Morvarid | 3 | 46° 58′ 08″ | 34° 52′ 33″ | 1850 |
| Isfahan | Kooshk | Kooshk | 13 | 32° 38′ 39″ | 51° 30′ 14″ | 1604 |
| Isfahan | Asgharabad | Asgharabad | 8 | 32° 39′ 02″ | 51° 29′ 24″ | 1607 |
| Markazi | Zarandeyeh | Vidar | 7 | 35° 19′ 22″ | 50° 06′ 55″ | 1688 |
| Markazi | Zarandeyeh | Vesmagh | 9 | 35° 18′ 16″ | 50° 04′ 05″ | 1798 |
| Markazi | Zarandeyeh | Bandamir | 13 | 35° 16′ 10″ | 50° 02′ 50″ | 1910 |
| Markazi | Zarandeyeh | Razghan | 10 | 35° 19′ 56″ | 49° 57′ 14″ | 1889 |
| Markazi | Zarandeyeh | Aliver | 8 | 35° 23′ 18″ | 49° 59′ 42″ | 1780 |
| Markazi | Saveh | Aghkahriz | 11 | 35° 06′ 09″ | 49° 48′ 01″ | 1545 |
| Markazi | Saveh | Dolatabad | 7 | 35° 06′ 24″ | 49° 48′ 34″ | 1557 |
| Markazi | Saveh | Kahak | 4 | 35° 04′ 54″ | 49° 48′ 46″ | 1508 |
| Markazi | Saveh | Hini | 10 | 35° 05′ 12″ | 49° 49′ 45″ | 1477 |
| Markazi | Khondab | Khondab | 11 | 34° 17′ 46″ | 49° 04′ 30″ | 1674 |

**Notes:** In the Kooshk area, 4 Tolaee, 2 Kaftarbache, and 7 Gonjooni genotypes were identified. In the Asgharabad area, 8 Gonjooni genotypes were identified. These names represent the local designations used for the genotypes in the respective areas. All samples collected from Isfahan and Markazi provinces belong to *Pyrus communis*, whereas those collected from Kurdistan province belong to *Pyrus syriaca*.

**S2 Table.** Cluster formation stages of pear genotypes.

| Genotypes | Stage | Distance | Cluster1 | Cluster2 | Number of Clusters | New Cluster | Next Stage |
| --- | --- | --- | --- | --- | --- | --- | --- |
| ‘Tolaee-1’ | 1 | 8.82 | 92 | 94 | 135 | 92 | 10 |
| ‘Tolaee-2’ | 2 | 9.68 | 2 | 4 | 134 | 2 | 41 |
| ‘Tolaee-3’ | 3 | 9.82 | 90 | 95 | 133 | 90 | 4 |
| ‘Tolaee-4’ | 4 | 10.32 | 88 | 90 | 132 | 88 | 33 |
| ‘Kaftarbache-1’ | 5 | 11.12 | 46 | 54 | 131 | 46 | 60 |
| ‘Kaftarbache-2’ | 6 | 11.34 | 55 | 82 | 130 | 55 | 19 |
| ‘Gonjooni-1’ | 7 | 12.16 | 64 | 79 | 129 | 64 | 18 |
| ‘Gonjooni-2’ | 8 | 12.45 | 5 | 6 | 128 | 5 | 99 |
| ‘Gonjooni-3’ | 9 | 12.68 | 112 | 126 | 127 | 112 | 30 |
| ‘Gonjooni-4’ | 10 | 12.68 | 89 | 92 | 126 | 89 | 33 |
| ‘Gonjooni-5’ | 11 | 12.96 | 48 | 81 | 125 | 48 | 24 |
| ‘Gonjooni-6’ | 12 | 12.99 | 122 | 128 | 124 | 122 | 40 |
| ‘Gonjooni-7’ | 13 | 13.01 | 130 | 134 | 123 | 130 | 114 |
| ‘Gonjooni-8’ | 14 | 13.04 | 38 | 39 | 122 | 38 | 86 |
| ‘Gonjooni-9’ | 15 | 13.14 | 58 | 87 | 121 | 58 | 48 |
| ‘Gonjooni-10’ | 16 | 13.40 | 56 | 74 | 120 | 56 | 76 |
| ‘Gonjooni-11’ | 17 | 13.41 | 24 | 28 | 119 | 24 | 57 |
| ‘Gonjooni-12’ | 18 | 13.42 | 64 | 108 | 118 | 64 | 23 |
| ‘Gonjooni-13’ | 19 | 13.42 | 55 | 86 | 117 | 55 | 64 |
| ‘Gonjooni-14’ | 20 | 13.44 | 22 | 65 | 116 | 22 | 37 |
| ‘Gonjooni-15’ | 21 | 13.70 | 50 | 83 | 115 | 50 | 24 |
| ‘Hini-1’ | 22 | 13.95 | 31 | 107 | 114 | 31 | 53 |
| ‘Hini-2’ | 23 | 14.02 | 64 | 78 | 113 | 64 | 39 |
| ‘Hini-3’ | 24 | 14.05 | 48 | 50 | 112 | 48 | 55 |
| ‘Hini-4’ | 25 | 14.09 | 44 | 96 | 111 | 44 | 44 |
| ‘Hini-5’ | 26 | 14.15 | 43 | 66 | 110 | 43 | 39 |
| ‘Hini-6’ | 27 | 14.37 | 114 | 118 | 109 | 114 | 73 |
| ‘Hini-7’ | 28 | 14.47 | 40 | 60 | 108 | 40 | 58 |
| ‘Hini-8’ | 29 | 14.65 | 20 | 109 | 107 | 20 | 62 |
| ‘Hini-9’ | 30 | 14.70 | 112 | 119 | 106 | 112 | 59 |
| ‘Hini-10’ | 31 | 14.80 | 129 | 133 | 105 | 129 | 49 |
| ‘Kahak-1’ | 32 | 14.88 | 19 | 26 | 104 | 19 | 69 |
| ‘Kahak-2’ | 33 | 15.04 | 88 | 89 | 103 | 88 | 50 |
| ‘Kahak-3’ | 34 | 15.33 | 67 | 68 | 102 | 67 | 54 |
| ‘Kahak-4’ | 35 | 15.39 | 11 | 12 | 101 | 11 | 99 |
| ‘Vesmagh-1’ | 36 | 15.44 | 113 | 131 | 100 | 113 | 59 |
| ‘Vesmagh-2’ | 37 | 15.52 | 22 | 85 | 99 | 22 | 51 |
| ‘Vesmagh-3’ | 38 | 15.63 | 69 | 71 | 98 | 69 | 77 |
| ‘Vesmagh-4’ | 39 | 15.80 | 43 | 64 | 97 | 43 | 64 |

**S2 Table.** Continued.

| ‘Vesmagh-5’ | 40 | 15.83 | 122 | 125 | 96 | 122 | 49 |
| --- | --- | --- | --- | --- | --- | --- | --- |
| ‘Vesmagh-6’ | 41 | 15.90 | 1 | 2 | 95 | 1 | 94 |
| ‘Vesmagh-7’ | 42 | 15.99 | 57 | 59 | 94 | 57 | 45 |
| ‘Vesmagh-8’ | 43 | 16.05 | 41 | 63 | 93 | 41 | 74 |
| ‘Vesmagh-9’ | 44 | 16.24 | 35 | 44 | 92 | 35 | 58 |
| ‘Dolatabad-1’ | 45 | 16.56 | 52 | 57 | 91 | 52 | 101 |
| ‘Dolatabad-2’ | 46 | 16.57 | 18 | 104 | 90 | 18 | 57 |
| ‘Dolatabad-3’ | 47 | 16.66 | 32 | 103 | 89 | 32 | 130 |
| ‘Dolatabad-4’ | 48 | 16.97 | 58 | 84 | 88 | 58 | 81 |
| ‘Dolatabad-5’ | 49 | 16.97 | 122 | 129 | 87 | 122 | 75 |
| ‘Dolatabad-6’ | 50 | 17.05 | 88 | 93 | 86 | 88 | 84 |
| ‘Dolatabad-7’ | 51 | 17.06 | 22 | 97 | 85 | 22 | 68 |
| ‘Aliver-1’ | 52 | 17.29 | 14 | 16 | 84 | 14 | 90 |
| ‘Aliver-2’ | 53 | 17.31 | 31 | 47 | 83 | 31 | 85 |
| ‘Aliver-3’ | 54 | 17.44 | 67 | 115 | 82 | 67 | 68 |
| ‘Aliver-4’ | 55 | 17.53 | 48 | 80 | 81 | 48 | 74 |
| ‘Aliver-5’ | 56 | 17.72 | 23 | 102 | 80 | 23 | 92 |
| ‘Aliver-6’ | 57 | 17.74 | 18 | 24 | 79 | 18 | 69 |
| ‘Aliver-7’ | 58 | 17.89 | 35 | 40 | 78 | 35 | 70 |
| ‘Aliver-8’ | 59 | 17.97 | 112 | 113 | 77 | 112 | 73 |
| ‘Vidar-1’ | 60 | 17.99 | 46 | 62 | 76 | 46 | 66 |
| ‘Vidar-2’ | 61 | 18.04 | 8 | 99 | 75 | 8 | 95 |
| ‘Vidar-3’ | 62 | 18.12 | 20 | 34 | 74 | 20 | 85 |
| ‘Vidar-4’ | 63 | 18.28 | 42 | 51 | 73 | 42 | 80 |
| ‘Vidar-5’ | 64 | 18.29 | 43 | 55 | 72 | 43 | 72 |
| ‘Vidar-6’ | 65 | 18.40 | 27 | 110 | 71 | 27 | 77 |
| ‘Vidar-7’ | 66 | 18.53 | 46 | 111 | 70 | 46 | 90 |
| ‘Aghkahriz-1’ | 67 | 18.97 | 120 | 132 | 69 | 120 | 93 |
| ‘Aghkahriz-2’ | 68 | 19.02 | 22 | 67 | 68 | 22 | 86 |
| ‘Aghkahriz-3’ | 69 | 19.14 | 18 | 19 | 67 | 18 | 82 |
| ‘Aghkahriz-4’ | 70 | 19.31 | 35 | 61 | 66 | 35 | 78 |
| ‘Aghkahriz-5’ | 71 | 19.42 | 100 | 135 | 65 | 100 | 95 |
| ‘Aghkahriz-6’ | 72 | 19.62 | 43 | 53 | 64 | 43 | 96 |
| ‘Aghkahriz-7’ | 73 | 19.63 | 112 | 114 | 63 | 112 | 91 |
| ‘Aghkahriz-8’ | 74 | 19.65 | 41 | 48 | 62 | 41 | 97 |
| ‘Aghkahriz-9’ | 75 | 19.68 | 122 | 123 | 61 | 122 | 91 |
| ‘Aghkahriz-10’ | 76 | 19.91 | 56 | 124 | 60 | 56 | 101 |
| ‘Aghkahriz-11’ | 77 | 19.93 | 27 | 69 | 59 | 27 | 107 |
| ‘Razghan-1’ | 78 | 20.21 | 35 | 76 | 58 | 35 | 104 |
| ‘Razghan-2’ | 79 | 20.46 | 21 | 29 | 57 | 21 | 125 |
| ‘Razghan-3’ | 80 | 20.48 | 42 | 117 | 56 | 42 | 103 |

**S2 Table.** Continued.

| ‘Razghan-4’ | 81 | 20.54 | 49 | 58 | 55 | 49 | 89 |
| --- | --- | --- | --- | --- | --- | --- | --- |
| ‘Razghan-5’ | 82 | 20.66 | 15 | 18 | 54 | 15 | 100 |
| ‘Razghan-6’ | 83 | 20.89 | 36 | 37 | 53 | 36 | 96 |
| ‘Razghan-7’ | 84 | 21.04 | 88 | 91 | 52 | 88 | 116 |
| ‘Razghan-8’ | 85 | 21.11 | 20 | 31 | 51 | 20 | 100 |
| ‘Razghan-9’ | 86 | 21.20 | 22 | 38 | 50 | 22 | 97 |
| ‘Razghan-10’ | 87 | 21.52 | 10 | 73 | 49 | 10 | 111 |
| ‘Bandamir-1’ | 88 | 21.53 | 7 | 72 | 48 | 7 | 102 |
| ‘Bandamir-2’ | 89 | 21.69 | 49 | 77 | 47 | 49 | 98 |
| ‘Bandamir-3’ | 90 | 21.78 | 14 | 46 | 46 | 14 | 104 |
| ‘Bandamir-4’ | 91 | 21.99 | 112 | 122 | 45 | 112 | 93 |
| ‘Bandamir-5’ | 92 | 22.03 | 13 | 23 | 44 | 13 | 121 |
| ‘Bandamir-6’ | 93 | 22.62 | 112 | 120 | 43 | 112 | 108 |
| ‘Bandamir-7’ | 94 | 22.63 | 1 | 3 | 42 | 1 | 131 |
| ‘Bandamir-8’ | 95 | 22.65 | 8 | 100 | 41 | 8 | 102 |
| ‘Bandamir-9’ | 96 | 22.77 | 36 | 43 | 40 | 36 | 106 |
| ‘Bandamir-10’ | 97 | 22.80 | 22 | 41 | 39 | 22 | 103 |
| ‘Bandamir-11’ | 98 | 23.17 | 49 | 105 | 38 | 49 | 111 |
| ‘Bandamir-12’ | 99 | 23.20 | 5 | 11 | 37 | 5 | 115 |
| ‘Bandamir-13’ | 100 | 23.53 | 15 | 20 | 36 | 15 | 105 |
| ‘Khondab-1’ | 101 | 23.68 | 52 | 56 | 35 | 52 | 106 |
| ‘Khondab-2’ | 102 | 24.05 | 7 | 8 | 34 | 7 | 109 |
| ‘Khondab-3’ | 103 | 24.42 | 22 | 42 | 33 | 22 | 110 |
| ‘Khondab-4’ | 104 | 24.63 | 14 | 35 | 32 | 14 | 105 |
| ‘Khondab-5’ | 105 | 25.49 | 14 | 15 | 31 | 14 | 117 |
| ‘Khondab-6’ | 106 | 25.77 | 36 | 52 | 30 | 36 | 118 |
| ‘Khondab-7’ | 107 | 25.94 | 27 | 75 | 29 | 27 | 113 |
| ‘Khondab-8’ | 108 | 26.06 | 112 | 116 | 28 | 112 | 112 |
| ‘Khondab-9’ | 109 | 26.61 | 7 | 98 | 27 | 7 | 115 |
| ‘Khondab-10’ | 110 | 26.62 | 22 | 45 | 26 | 22 | 120 |
| ‘Khondab-11’ | 111 | 26.96 | 10 | 49 | 25 | 10 | 126 |
| ‘Kamyaran-1’ | 112 | 27.74 | 112 | 127 | 24 | 112 | 114 |
| ‘Kamyaran-2’ | 113 | 27.93 | 25 | 27 | 23 | 25 | 124 |
| ‘Kamyaran-3’ | 114 | 28.43 | 112 | 130 | 22 | 112 | 116 |
| ‘Kamyaran-4’ | 115 | 28.91 | 5 | 7 | 21 | 5 | 126 |
| ‘Kamyaran-5’ | 116 | 29.00 | 88 | 112 | 20 | 88 | 119 |
| ‘Kamyaran-6’ | 117 | 29.21 | 14 | 17 | 19 | 14 | 124 |
| ‘Kamyaran-7’ | 118 | 29.39 | 36 | 70 | 18 | 36 | 121 |
| ‘Kamyaran-8’ | 119 | 29.92 | 88 | 106 | 17 | 88 | 127 |
| ‘Kamyaran-9’ | 120 | 30.07 | 22 | 30 | 16 | 22 | 123 |
| ‘Kamyaran-10’ | 121 | 30.47 | 13 | 36 | 15 | 13 | 122 |

**S2 Table.** Continued.

| ‘Kamyaran-11’ | 122 | 31.15 | 13 | 121 | 14 | 13 | 123 |
| --- | --- | --- | --- | --- | --- | --- | --- |
| ‘Kamyaran-12’ | 123 | 32.02 | 13 | 22 | 13 | 13 | 128 |
| ‘Marivan-1’ | 124 | 32.03 | 14 | 25 | 12 | 14 | 125 |
| ‘Marivan-2’ | 125 | 33.75 | 14 | 21 | 11 | 14 | 129 |
| ‘Marivan-3’ | 126 | 34.27 | 5 | 10 | 10 | 5 | 128 |
| ‘Marivan-4’ | 127 | 35.94 | 88 | 101 | 9 | 88 | 131 |
| ‘Marivan-5’ | 128 | 37.14 | 5 | 13 | 8 | 5 | 132 |
| ‘Marivan-6’ | 129 | 38.50 | 14 | 136 | 7 | 14 | 133 |
| ‘Marivan-7’ | 130 | 40.12 | 32 | 33 | 6 | 32 | 134 |
| ‘Marivan-8’ | 131 | 46.48 | 1 | 88 | 5 | 1 | 132 |
| ‘Marivan-9’ | 132 | 48.67 | 1 | 5 | 4 | 1 | 135 |
| ‘Marivan-10’ | 133 | 53.99 | 9 | 14 | 3 | 9 | 134 |
| ‘Marivan-11’ | 134 | 58.14 | 9 | 32 | 2 | 9 | 135 |
| ‘Marivan-12’ | 135 | 65.85 | 1 | 9 | 1 | 1 | -- |
| ‘Marivan-13’ | 136 | - | - | - | - | - | - |

Distance type: Euclidean.
